# How Similar Are Students’ Aggregated State Emotions to Their Self-Reported Trait Emotions? Results from a Measurement Burst Design Across Three School Years

**DOI:** 10.1007/s10648-025-09995-1

**Published:** 2025-03-13

**Authors:** Melanie M. Keller, Takuya Yanagida, Oliver Lüdtke, Thomas Goetz

**Affiliations:** 1 Department of Physics Education, IPN–Leibniz Institute for Science and Mathematics Education, Olshausenstrasse 62, 24118 Kiel, Germany; 2 Department of Developmental and Educational Psychology, University of Vienna, Vienna, Austria; 3 IPN–Leibniz Institute for Science and Mathematics Education, Center for International Student Assessment, Kiel, Germany

**Keywords:** Emotions, States, Traits, Aggregation

## Abstract

Students’ emotions in the classrom are highly dynamic and thus typically strongly vary from one moment to the next. Methodologies like experience sampling and daily diaries have been increasingly used to capture these momentary emotional states and its fluctuations. A recurring question is to what extent aggregated state ratings of emotions over a longer period of time are similar to self-reported traits of emotions. Thus, this study aims to investigate the extent of similarity between students’ aggregated emotional states and self-reported traits over a two-week period in three consecutive school years (*N*_T1_ = 149, average age_T1_ = 15.64 years). Six discrete emotions (enjoyment, anger, pride, anxiety, shame, and boredom) were assessed in German, English, French, and mathematics classes. We investigated similarity in terms of convergence, mean-level differences, long-term stability, and incremental predictive validity of aggregated states and self-reported traits. Results indicated substantial convergence between aggregated states and self-reported traits, with both showing similar long-term stability. However, aggregated states did not demonstrate superior predictive validity compared to self-reported traits for academic outcomes, while momentary assessments offer insights into short-term emotional fluctuations, on a person-aggregated level aggregated states and self-reported traits behave rather similarly. This suggests that both can be used interchangeably to study students’ trait-related research questions, like interindividual differences or long-term emotional processes in educational settings.

**Supplementary Information:**

The online version contains supplementary material available at 10.1007/s10648-025-09995-1.

## Introduction

Emotions are highly dynamic phenomena. Also school students’ emotional experiences in the classroom, like their feelings of enjoyment, pride, anger, or anxiety, typically strongly vary from one moment to the next (Ahmed et al., 2010). In order to capture these momentary experiences (i.e., emotional states) and their fluctuations, studies in the recent years have increasingly employed corresponding methodologies such as experience sampling (ESM, e.g., Goetz et al., 2024), lesson diaries (e.g., Tam et al., 2019) or daily diaries (e.g., Karwowski et al., 2021). Still, despite the rising number of studies making full use of such data and tracing emotional dynamics (for an overview of analysis apporaches, see Bolger & Laurenceau, 2013; McNeish & Hamaker, 2020), a number of studies analyze data as aggregated states and on a person-level, drawing conclusions as to interindividual differences (Goetz et al., 2013, 2015; Ketonen et al., 2018; Martin et al., 2020; Moeller et al., 2020).

However, analyses on emotions on a person-level have typically been based on traits. Whereas emotional states capture an individual’s situation-specific emotional response to a stimulus (e.g., anxiety as experienced while working on a math test), emotional traits are understood as the individual’s tendencies to respond to certain situations with certain emotions (e.g., typical level of anxiety during math tests). One can argue that repeatedly capturing the situation-specific manifestation of an emotional state (“how strongly do you experience anxiety at this moment?”) infers the emotional trait (typical experiences of anxiety). While these aggregated emotional states seem to reflect generalized, one-time emotional trait reports regarding well-being (Brose et al., 2013), it remains empirically quite unclear to what extent aggregated states of discrete emotions resemble self-reported traits for instance regarding long-term stability, or predictive power with regard to relevant outcomes. Demonstrating similarities of aggregated emotional states to their respective self-reported traits is crucial for guiding future research and drawing conclusions for two main reasons. Firstly, aggregated states are discussed as equally valid or even superior to self-reported traits (see for example Beal, 2015). This is because aggregated states may mitigate some of the inherent drawbacks of global trait self-reports, such as memory biases or overreliance on self-schema. Secondly, one-time self-reported traits are more economical than multiple state assessments. Therefore, substituting self-reported traits with state assessments should only be considered when absolutely necessary and reasonable.

Previous research on the similarity between aggregated states and self-reported traits has primarily focused on personality (e.g., Breil et al., 2022) or affect (e.g., Röcke et al., 2011), with most studies conducted outside the classroom context. However, discrete emotions are more strongly tied to situational stimuli than affect. And students’ emotions in the classroom are influenced by a range of situational factors such as the day of the week (Cranford et al., 2006), school subjects and subject matter (Goetz et al., 2007), students’ interest or level of achievement in a subject (Pekrun et al., 2017), peer interactions and classroom characteristics such as classroom climate (Becker-Kurz & Morris, 2015), learning activities and instruction (Bieg et al., 2017), as well as teacher characteristics (Becker et al., 2014) and student–teacher relationships (Goetz et al., 2021). Further, previous studies comparing aggregated states with self-reported traits of discrete emotions have primarily focused on specific similarity criteria, such as the memory experience gap (e.g., Villinger et al., 2021) or the incremental prediction of outcomes (e.g., Krannich et al., 2022). However, comparisons of long-term stability have been notably lacking.

In the present study we aim to compare the extent of similarity between two trait emotion measures—students’ averaged (i.e., person-aggregated) emotional states and their emotional self-reported traits—using several criteria for similarity. To achieve this, we conducted a measurement burst study (Sliwinski, 2008), capturing students’ emotional states in the classroom over two weeks using ESM across three consecutive school years, with self-reported emotional traits assessed each year. Regarding similarity, we first determine *convergence* between aggregated states and self-reported traits by examining their correlation; second, we examine *mean-level differences* of aggregated states and self-reported traits; third, we investigate *differences in long term stability* (one year span and two year span); finally, we explore predictive power of aggregated states above and beyond self-reported traits and vice versa, that is, their *incremental predictive power with regard to achievement, intrinsic value, self-concept, and career orientation*.

By incorporating multiple similarity criteria, this study expands the existing literature by focusing on students’ discrete pleasant and unpleasant emotions in the classroom. Additionally, it is designed to capture a substantial portion of students’ classroom experiences, spanning multiple subjects and consecutive school years.

## Theoretical Background

### Students’ Emotional Experiences in School

Human life is full of emotions, and unsuprisingly, classrooms in schools are also very emotional places. Students experience a wide range of emotions where enjoyment and boredom appear the most frequent (e.g., Lichtenfeld et al., 2012; Peixoto et al., 2015; Pekrun et al., 2011).

Emotions in the classroom are closely linked to achievement and learning situations. They arise during various learning activities, social interactions with teachers or peers, and are often tied to specific content or the academic domain (Pekrun, 2006). The Control Value Theory of Achievement Emotions (CVT) (Pekrun, 2006) is a prominent theory describing precursors and consequences of students’ emotions. Over the past approximately 15 years, numerous studies have consistently supported the fundamental principles of this framework. For example, research has highlighted the significance of emotions in various outcomes, such as students’ achievement, (e.g., Pekrun et al., 2017), as well as as their motivation, for example as students’ self-concept (e.g., van der Beek et al., 2017) or interest (e.g., Schukajlow & Rakoczy, 2016). Furthermore, emotions have been found to influence students’ career orientation and choices (e.g., Ahmed & Mudrey, 2019; Kyttälä & Björn, 2010).

### Emotional States and Traits

#### Defining States and Traits

Since its inception, psychological research has focused on predicting how humans will react or behave in a specific situation by decribing and classifying their personality structure. Thus, the distinction between personal and situational characteristics, along with the question of which exerts a stronger influence on current behavior, was already ingrained in the foundations of this research from its outset. Over decades, the pendulum in the person-situation debate (see Epstein & O'Brien, 1985) has swung back and forth between favoring the influence of personal traits and emphasizing the power of situational factors. Foundational personality psychologists including Allport (1927), Eysenck (1947) and Cattell (1946) postulated the existence of stable personality traits. However, the failure to find substantial correlations between such traits and momentary behaviors led to a crisis in personality psychology in the 1960s, culminating in Mischel’s (1968) publication which dismissed the possibility of stable traits altogether due to lack of evidence.

An explanation for and possible solution to the crisis was proposed in a series of publications by Epstein (e.g., 1979, 1980). He posits that “the existence of traits is the demonstration of cross-situational stability” and that the existence of “relatively broad, stable response dispositions, or traits, does not conflict with the assumption that situations often exert a strong influence on behavior” (Epstein, 1979, p. 1122).^1^ In light of variations in human responses due to situational factors, he recommends collecting momentary responses from a wide array of situations and time points to identify correlations with stable traits. This resolution suggests that there are often two sides to one construct such as discrete emotions, namely a state and a trait side.

When distinguishing states from traits, Fridhandler (1986) uses the terms “occurrences” and “dispositions” (p. 171). Emotional states defined as momentary occurrences describe transient emotional experiences (see Eid et al., 1999), whereas traits including emotional traits are defined as generalized and lasting predispositions to react to certain situations in a consistent way (see for instance Endler & Kocovski, 2001). Traits are characterized by their consistency across situations and stability over time (see Fajkowska & Kreitler, 2018). Because of their consistency, traits may be inferred by repeated observations across situations (these traits are sometimes also called “abstract traits”, see Pekrun, 1988, p. 23). The diversity of situations can vary based on the level of generality of the traits. For instance, if someone is repeatedly observed being anxious in achievement, work-related, and social situations, this may indicate a more generalized trait of anxiety. In contrast, if the observation is limited to achievement-related situations, the person would be characterized as having achievement-related anxiety. However, while traits describe tendencies to react in specific ways, their manifestation in any given situation depends on situational cues; thus, their consistency across situations and stability across time cannot be assumed to be absolute.

According to Conner and Barrett (2012), traits and states represent different aspects about the self; they distinguish between “the experiencing (momentary), remembering (retrospective), and believing (trait) self” (p. 4). The function of emotional traits is to “filter[s] and consolidate[s] our experiences so that we can learn, communicate, and make decisions about the future based on our past” and thus to “maintain [an individual’s] identity through time” (Conner & Barrett, 2012, p. 5). In contrast, emotional states serve to provide immediate feedback about one’s environment, guiding real-time responses and adaptations to current situations.

#### Methodological Considerations Regarding the Measurement of States and Traits

The above mentioned crisis in personality psychology and the ensuing person-situation debate were essentially conceptual in nature: is there such a thing as stable traits, or do situational influences reign supreme? However, the possible solution proposed by Epstein (1980) was methodological in nature: aggregating over several instances to infer traits or to establish correlations with (other assessments of) traits (this approach was already suggested by Allport, 1931). As such, conceptual and measurement issues have always blended into one another.

Indeed, states and traits are often defined co-dependently. For instance, the whole trait theory postulates traits as density distributions of states (Fleeson & Jayawickreme, 2015), and states result from interactions of the person and the situation at a specific time point (a similar argument is also provided for state anxiety by Endler & Kocovski, 2001). Similarly, latent state trait theory decomposes any observed score into a latent state and measurement error, whereby the latent state also includes a latent trait component which captures the stable part across the state measures of one person (e.g., Geiser et al., 2017; Steyer et al., 2015). Just as latent state trait theory thus considers traits as latent variables tangible only through their multiple manifestations as states, Fridhandler (1986) also asserts that traits can ever only be inferred by using a large number of occasions.

Acknowledging the state-trait distinction, an increasing number of studies employ methodology such as experience sampling or diary methods to assess state emotions as experienced in a concrete situation, thus separating them from self-reported traits in terms of assessment. Because of this rapid proliferation of state assessments, it becomes a relevant question how similar aggregated state emotions are compared to global self-reports of trait emotions.

Over the last three decades, many studies have examined how high the person-specific component is in state reports of personality, affect, or discrete emotions. Thereby, evidence clearly supports the idea of a person-specific component in individuals’ momentary states denotative of an emotional trait. For example and employing a measurement-burst design, Röcke et al. (2011) determined that 34% of variance was on the momentary and 47% on the person-level for positive affect (the remaining percentages of variance were on the burst-level), and for negative affect around 50% was on the momentary and 30% on the person-level. Similarly, Rauthmann et al. (2018) reported intraclass correlations (ICCs) between 0.29 and 0.46 for six personality dimensions, evidencing that about a third to half of the variance in state personality lies on the person-level. In a study focusing on emotional experiences in personal (as opposed to technology-mediated) interactions, Petrova and Schulz (2022) present ICCs of 0.36 and 0.44 for happiness and anxiety, respectively, also supporting the idea that discrete momentary emotions have a person-specific component.

These findings also extend to the educational context and students’ classroom emotions. When students repeatedly reported on their momentary emotions in the classroom, 32% of variance was on the person-level for anxiety, 25% regarding enjoyment, 23% regarding frustration, and 21% regarding boredom (Study 2 in Moeller et al., 2020). This person-specific variance was somewhat higher when students retrospectively evaluated their emotions in class regarding the past lesson: here, 30% of variance was on the person-level for anxiety, 40% for boredom, 41% for anger, 49% for enjoyment, 50% for shame, and 59% for pride (Ahmed et al., 2010). When applying latent state trait modeling instead of multilevel modeling, about a third of variance lay on the person-level for students’ emotions when assessed retrospectively for the past lesson (Nett et al., 2017) and about 50% regarding their momentary classroom emotions (Respondek et al., 2019). Whether these findings extend beyond classroom emotions to students’ test-related emotions remains unclear. Despite extensive research on test anxiety, we are not aware of any study that uses aggregated state test anxiety scores to compare with self-reported traits. In total, these findings lend credence to the idea that when students’ momentary emotional experiences, i.e., their state emotions, are measured repeatedly, the common variance in these emotional states captures a person-specific, trait-like emotion component. Aggregating several emotional states to infer an emotional trait therefore seems plausible.

Using aggregated emotional states as measurement of emotional traits appears a reasonable solution to the known biases in single-occasion, retrospective and generalized measures of traits. Global emotional trait reports are influenced by the momentary emotional state at the time of the assessment (Brose et al., 2013). Self-reported traits are also susceptible to memory biases which commonly result in the so-called memory-experience gap (e.g., Moeller et al., 2020; Neubauer et al., 2020; Wirtz et al., 2003). These memory errors make people seem incapable, or at least unreliable, when it comes to evaluating their emotional experiences in retrospect. Robinson and Clore (2002) see the reason for this inability in the fact that while momentary experiences are encoded in the episodic memory, this type of memory declines rapidly over time. Thus, when asked to retrospectively evaluate their emotional experiences, individuals draw on their semantic knowledge because the experience itself is no longer immediately accessible (see also Levine et al., 2009).

Thus, when people cannot accurately recall their emotional experiences, measuring their momentary experiences and summarizing them into a trait score as an alternative to retrospective single-occasion trait reports seems to be a viable option and is sometimes even thought to be superior to global single-occasion self-reports (Beal, 2015). To what extent, however, are such aggregated states comparable to self-reported traits?

### Empirical Evidence Regarding Similarity Between Aggregated States and Self-Reported Traits

Studies examining similarity between aggregated states and self-reported traits utilize various criteria. Typically, three main criteria are employed: correlation between aggregated states and self-reported traits (using different terms when investigating this type of similarity, e.g., correspondence convergence: Neubauer et al., 2020; convergent validity: Rauthmann et al., 2018; correspondence: Röcke et al., 2011); mean-level differences often called the memory experience gap (Miron-Shatz et al., 2009; Neubauer et al., 2020; Wirtz et al., 2003); and prediction of a set of correlates (Rauthmann et al., 2018; Wirtz et al., 2003). Additionally, the theoretical criterion temporal stability suggests that states are transient while traits are enduring (Fridhandler, 1986), but we are not aware of any study in which this criterion has been applied to comparing aggregated states to self-reported traits regarding discrete emotions, perhaps because of the complex and labor-intensive study design an investigation of long-term stability of aggregated states would require. In our study, we integrate all four criteria to assess similarities between aggregated states and self-reported traits, encompassing convergence (i.e., correlations), mean-level differences, temporal stability, and incremental predictive validity of a set of external criteria.

#### Convergence Between Aggregated States and Self-Reported Traits

Substantial but imperfect convergence between aggregated states and self-reported traits is indeed demonstrable for a wide range of emotional constructs and across contexts. For instance, Finnigan and Vazire (2018) reported zero-order correlations ranging from 0.37 to 0.55 between aggregated state and self-reported traits of personality factors. A more heterogeneous but overall similar convergence is reported by Rauthmann et al. (2018) using various personality measures and a nomological approach to determine convergence. Regarding affective experiences, Wirtz et al. (2003) investigated students’ momentary and retrospective (2–4 days and 4 weeks after) positive and negative affective holiday experiences: zero-order correlations between aggregated states and retrospective ratings thereby ranged from 0.53 to 0.70. A similarly good but not perfect convergence is reported by Röcke et al. (2011) for older adults and between momentary affect ratings across one year and retrospective end-of-year rating, with zero-order correlations being 0.63 for positive and 0.55 for negative affect. In an attempt to distinguish good from bad days rather than happy from unhappy individuals, Neubauer et al. (2020) investigated within-person convergence between aggregated states and self-reported traits and—similar to the between-person findings listed above—also found substantial but imperfect convergence for positive (*r* = 0.63) as well as negative affect (*r* = 0.60); additionally, the authors aggregated individuals’ affective states as well as their self-reported traits and found near-perfect convergence (*r* = 0.97 for both positive and negative affect). Brose et al. (2013) also investigated convergence within a person and found that the affective states assessed several times a day contribute significantly to the ratings of well-being at the end of the day. Overall, findings from previous studies support the theoretical considerations (Conner & Barrett, 2012; Robinson & Clore, 2002) by demonstrating substantial but imperfect convergence between aggregated states and traits.

However, the above mentioned studies focus on affective experience rather than discrete emotions. Compared to emotions, affect is not necessarily tied to a specific stimulus and affective episodes, including moods, typically last longer than emotional events. As a result, affect tends to be less fluctuating than emotions which are more strongly tied to situational stimuli and events. Subsequently, aggregated states in affect would have higher shares with self-reported traits compared to discrete emotions. Presently and for discrete emotions, investigations systematically comparing aggregated states to self-reported traits seldom focus on or provide information about convergence. For instance, Barnes et al. (2002) focused on reliability issues regarding the State Trait Anxiety Inventory, but did not quantify the relation between state and trait anxiety. Pekrun et al. (2017) in developing the Epistemically-Related Emotion Scales found convergence between emotional states aggregated over three text-reading activities and emotions assessed once retrospectively for all reading activities; their correlations ranged between 0.65 and 0.83. Some studies investigating students’ emotions in the classroom and applying state and trait measures report correlations between aggregated states and self-reported traits for selected discrete emotions (e.g., correlations of aggregated state and trait boredom of around r = 0.50: Krannich et al., 2022).

Findings regarding convergence for discrete emotions are scattered across a wide range of publications which focus on very diverse research questions, making information on convergence difficult to summarize. However, drawing on the findings available based on affect and discrete emotions (e.g., Endler et al., 1994), it appears that substantial but imperfect convergence between aggregated states and self-reported traits of affect also extends to discrete emotions.

#### Mean-Level Differences Between Aggregated States and Self-Reported Traits

The memory-experience gap has been shown for a range of affective constructs. Most famous are the studies regarding experienced and remembered pain (e.g., Redelmeier & Kahneman, 1996). Beyond that, the gap has also been shown for instance for post-traumatic stress disorder symptoms (Greene et al., 2022) and positive and negative affect in the context of depression (Rinner et al., 2019), but also everyday positive and negative affective experiences (Neubauer et al., 2020; Tadić et al., 2014; Villinger et al., 2021). Thereby, the memory-experience gap is systematic in that it seems larger for negative compared to positive affective experiences (Miron-Shatz et al., 2009; Neubauer et al., 2020) and the gap becomes smaller for older individuals evaluating their negative affect (Junghaenel et al., 2021).

In the school context, a few studies have corroborated the memory-experience gap in students for the discrete emotion of anxiety in mathematics (Goetz et al., 2013), but also for students’ enjoyment, pride, and anger (Bieg et al., 2014). Teachers’ emotional experiences in the classroom and relating to teaching also evidence the memory-experience gap (Goetz et al., 2015). Generally, the evidence across those studies does not allow for a systematic conclusion regarding mean-level differences as investigations often focused on a limited number of or even only one academic emotion or domain. Based on previous evidence, however, as well as evidence outside of the school context, we can assume to find the memory-experience gap for the present study in a way that students’ self-reported traits of emotions should be rated higher than their aggregated emotional states.

#### Long-Term Stability of Aggregated States and Self-Reported Traits

Based on the definitions of states vs. traits, it seems trivial to investigate the stability of emotional states compared to traits over time. *Aggregated* emotional states, however, capture the common variance in a number of states over time attributed to stable person-specific factors (called consistency in the language of latent state trait theory, Steyer et al., 2015). Temporal stability of aggregated states should thus be higher than that of one single momentary state; however, it remains unclear how aggregated states compare to self-reported traits in terms of stability.

Only a few studies explicitly compare long-term stabilities of aggregated states to that of self-reported traits (as most studies comparing states to traits do not use aggregated states). For depressive symptoms and self-esteem, the results of Braun et al. (2021) support the assumption of less stable aggregated states compared to self-reported traits.

For students’ classroom emotions we know that their discrete trait emotions remain relatively stable across the span of one school year (Forsblom et al., 2022; Mata et al., 2022; Pekrun et al., 2017). We are not aware of similar evidence of stability over time for aggreated state emotions. However, as aggregated affective states are comparatively volatile during adolescence (e.g., Larson et al., 2002; Maciejewski et al., 2015) we assume that students’ aggregated state emotions might be less stable across longer time frames than self-reported traits of emotions.

#### Predictive Validity of Aggregated States and Self-Reported Traits

Evidence outside of the school context supports the greater predictive power of self-reported traits vs. aggregated states for behavioral intentions (Wirtz et al., 2003), as well as the diminished power of actual experiences regarding future decisions (Finn, 2010; Kahneman et al., 1993; Redelmeier et al., 2003).

In the school context, ample evidence suggests students’ emotions are linked to important educational outcomes such as learning and achievement (see for instance Camacho-Morles et al., 2021; Pekrun et al., 2017; Valiente et al., 2012) or motivation (see Huang, 2011; Li et al., 2021). While this relationship seems consistent and robust for students’ self-reported traits of emotions, the evidence for students’ aggregated state emotions and how they relate to academic outcomes is sparse. Evidence exists regarding aggregated state emotions being related to trait motivational and learning related outcomes (epistemic emotions related to task value and learning strategies, Pekrun et al., 2017; enjoyment but not boredom related to mathematics interest, Schukajlow, 2015) as well as to students’ well-being (Ketonen et al., 2018). We are not aware of further studies that explicitly compare self-reported traits to aggregated state emotions and how they differentially relate to students’ outcomes. We know of only one study finding that students’ self-reported trait of boredom more strongly predicted students’ career aspirations than their aggregated state boredom (Krannich et al., 2022).

To summarize, similarity between aggregated states and traits is typically examined in terms of convergence (i.e., correlation), mean-level differences, long-term temporal stability, and predictive validity. While substantial convergence exists between these constructs, previous research indicates that aggregated states are less predictive of outcome criteria and less prominent compared to traits, although the latter is predominantly investigated outside educational contexts. Research on long-term stability comparing aggregated states to traits is largely absent, leaving unanswered questions about substitutability when studying students' emotional experiences over time.

## Aims and Hypotheses of the Present Research

The aim of the present study was to investigate the extent to which aggregated states of students’ emotions are similar or dissimilar to self-reported traits of emotions. In order to test similarity, we used data from a measurement burst study conducted in the German speaking part of Switzerland: students were followed and reported on their momentary classroom emotions (each year over the course of ten consecutive school days) and their trait emotions once each year from grades 9 through 11.

In this study, we consider students’ trait emotions as abstract dispositions and habitual emotional experiences in the classroom. Therefore, our use of the term “trait” may be unconventional, as we consider traits at a lower level of generality than is often the case, with a narrower range of situations and shorter time frames compared to lifelong dispositions. Six discrete emotions were assessed based on frequency and academic relevance: enjoyment and boredom are activity-related emotions and the most frequently experienced emotions in the classroom (Pekrun et al., 2011); anxiety is a prospective outcome-related emotion highly detrimental to achievement (Barroso et al., 2021); pride, shame, and anger are retrospective outcome-related emotions relevant for future effort and engagement (Pekrun & Linnenbrink-Garcia, 2012). In order to cover a relevant slice of students’ classroom experiences, the study covered four subjects in which the assessments took place, namely, German, English, French, and mathematics lessons which amounted to about 40 percent of students’ lesson time.

Synthesizing previous research on the similarity of (aggregated) states and self-reported traits, we gauged similarity based on their convergence, mean-level differences, long-term stability and predictive validity.

- **Hypothesis 1:** *convergence.* We investigated the correlation between aggregated states and self-reported traits for each discrete emotion to gauge convergence. We expected substantial but well below perfect correlations, suggesting imperfect convergence.
- **Hypothesis 2:** *mean-level differences.* We investigated mean-level differences between aggregated states and their respective self-reported trait counterparts. We expected students’ self-reported traits to rate higher than their aggregated states, particularly so for unpleasant emotions.
- **Hypothesis 3:** *stability.* We examined how stable aggregated states and self-reported traits are over a longer time frame by determining their respective auto-correlations across one-year and two-year spans. We expected higher auto-correlations of self-reported traits compared to that of aggregated states.
- **Hypothesis 4:** *predictive validity.* Finally, we examined how strongly aggregated states corrected by self-reported traits and vice versa correlated with external academic criteria (grades, intrinsic value, academic self-concept, and career orientation). The outcome criteria were chosen because they have been shown to be relevant in the educational context, evidencing links to students’ learning, achievement, course selection, and career choices. We expected predictive validity of self-reported traits over and above predictive validity of aggregated states.

## Methods

### Study Design

**Fig. 1:**
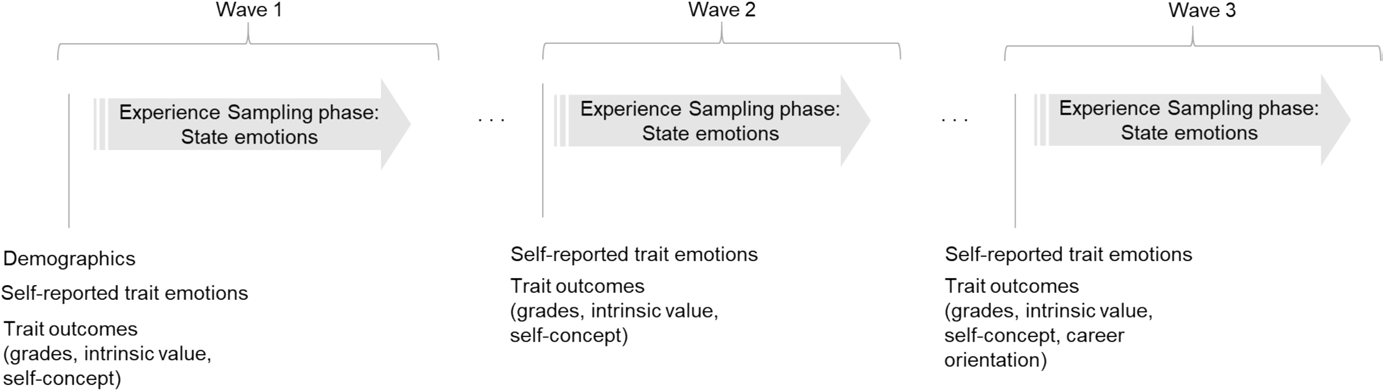
Design of the longitudinal measurement burst study. Each experience sampling phase lasted about ten school days during which state emotions were collected during German, English, French, and Mathematics classes. Data collection for each wave was conducted during the spring semester, spacing the waves roughly one school year apart from one another

The data for the present study came from a longitudinal measurement burst study across three school years. Parts of the study data have been used in previous publications with regard to different research questions, none of which focused on similarity between aggregated emotional states and traits (Becker et al., 2014; Bieg et al., 2017; Costache et al., 2021, 2022; Goetz et al., 2016, 2020, 2021, 2023; Krannich et al., 2019; Sticca et al., 2017, 2023; Wolff et al., 2021). The study was conducted in German-speaking Switzerland in eight schools from the high-achieving track in the Swiss educational system between 2012–2015. *N* = 161 students were randomly chosen from 44 different 9th grade classes (three to four students per class); these students were then followed over the course of three years up to grade 11. Students reported their domain-specific (German, English, French, and mathematics) trait emotions (enjoyment, anger, pride, anxiety, shame, and boredom) (for an overview of the assessments, see Fig. 1) once each year in the spring term. Their state emotions were assessed using experience sampling and iPod Touch 4G devices programmed with experience-sampling-software (iDialog Pad; Kubiak & Krog, 2012). Sampling was a combination of event- and time-sampling: Students activated the devices at the beginning of German, English, French, and mathematics lessons. The devices randomly signaled once 10–35 min after activation, prompting students to report their momentary emotional experiences. Each experience sampling phase in the three assessment waves lasted ten consecutive school days.

### Sample

In total, *N* = 161 students participated in the ESM assessment as well as in the trait assessment in at least one wave and were thus included in the present study. The students and measurement points were distributed as following across the waves: *N* = 149/122/106 students participated in the ESM assessments as well as in the trait assessments in waves 1, 2, and 3, respectively. These students provided *N* = 2668/1543/1258 state datapoints for the respective waves; the response rates, i.e., relation of number of responses to numbers of times the device was activated, were 81%, 80%, and 76%. For the three waves, 54/57/53% of students were female and students’ ages were on average 15.64/16.62/17.63 years (*SD* = 0.62/0.58/0.59 years).

### Missing Data

Our dataset included a total of 254 variables, resulting from the number of constructs, items, waves, and domains, for example: 6 (emotions) × 1 (item) × 4 (domains) × 3 (waves) = 72 as the number of variables for state emotions. The percentage of missing values across the 254 variables ranged from 9.32 to 42.86%. In total, 26.22% of data were missing, resulting from 32 students participating in only two waves of measurement, 45 students who participated in only one wave, and 40 students who participated in three waves, but had a general missing data pattern with missing values scattered throughout the entire data matrix.

Multiple imputation (Rubin, 1987) based on a fully conditional specification under the missing at random assumption was used to handle missing data. Predictors for the imputation model for each variable were selected based on the procedure described by van Buuren et al. (1999), using a minimum threshold of 0.25. On average, 39.69 predictors (*SD* = 12.51, Min = 11, Max = 75) were selected for the imputation model. Missing values were predicted using the predictive mean matching (PMM) algorithm (van Buuren, 2018) resulting in 100 multiply imputed data sets. Convergence check was conducted by inspecting R-hat convergence diagnostic for the mean and variance estimates. All R-hat measures were below 1.1 (*M* = 1.01/1.01 and *SD* = 0.01/0.01 for the mean and variance estimate), indicating convergence for all imputed variables.

### Instruments

Since experience sampling relies on self-reports in a given situation, the assessment of constructs needs to balance reliability and validity of measures with intrusiveness of the assessment. Thus and in line with previous studies, we chose to assess state emotions with single items (for a comparison between single- and multi-item measures of emotions, see Gogol et al., 2014). In an effort to eliminate differences in aggregated state and self-reported traits of emotions that are inherent to instruments and not the assessment method per se (i.e., using aggregated state or self-reported traits), we chose to also assess trait emotions using single item measures. As students’ academic emotions are organized in a domain specific way (see for example Goetz et al., 2006), trait and state emotions were assessed pertaining to the respective subject domains (i.e., German, Englisch, French, and Mathematics).

#### State Emotions

The assessment of students’ state achievement emotions was based on the class-related emotions in the Academic Emotion Questionnaire (AEQ, Pekrun et al., 2011), an established instrument widely used in previous research and adapted inside and outside the European context (e.g., Trigueros & Aguilar-Parra, 2022; Zhou & Wu, 2021). State emotions were assessed using single items. Item wordings were adapted where necessary to reflect momentary emotional experiences and included the main emotion-word for the assessment of enjoyment, anger, pride, anxiety, shame and boredom (e.g., “At the moment, I am angry” for state anger). Thus, six emotion items were used in each of the four academic domains (i.e., German, Englisch, French, and Mathematics). Item wordings can be found in the Appendix. All items were rated on a five-point Likert scale ranging from (1) *not at all* to (5) very *strongly*.

#### Trait Emotions

The assessment of trait emotions was also based on the class-related emotions in the AEQ. The emotions of enjoyment, anger, pride, anxiety, shame, and boredom were each assessed with respect to the four academic domains of German, Englisch, French, and Mathematics resulting in 24 trait emotion items. A sample item is “In German classes, I am usually angry” for trait anger in German. Items were rated on a five-point Likert scale ranging from (1) *not at all* to (5) *very strongly*. Trait emotions were assessed at the end of each experience sampling phase (see Fig. 1).

#### Trait External Criteria

To investigate incremental predictive validity of aggregated states corrected for self-reported traits and vice versa, we assessed students’ subject-specific grades, intrinsic value, self-concept, and approach and avoidance career orientation. These criteria were assessed at the beginning of each wave's experience sampling phases (see Fig. 1). All items (except grade) were rated on a five-point scale from (1) *strongly disagree* to (5) *strongly agree*. Item wordings for all items are shown in the Appendix.

##### Grades

Due to time constraints that did not allow administering an achievement test for each subject in each wave, we chose to gauge students’ academic achievement via grades as they are assumed to be an adequate measure of students’ academic achievement in a given domain (Canfield et al., 2015). For the present study, grades were assessed in the subject domains of German, English, French, and Mathematics as issued to students on their last report card (usually the half-term report card around January or February), with a sample item for German being “Which grade did you receive in German in your last report card?” Students provided a value between (1) *insufficient* and (6) *outstanding*.

##### Intrinsic Value

Intrinsic value reflects the personal relevance derived from the inherent qualities of a subject or an activity. It is close to academic interest which has been shown to be an influential disposition in motivational adaptation processes or for academic achievement (see for instance Wigfield & Cambria, 2010). Contrary to interest, however, value excludes the affective component inherent to common conceptualizations of interest and which overlaps with the emotion of enjoyment (see for instance, Krapp & Prenzel, 2011) which was why we chose intrinsic value instead of interest as an external criterion for the present study. Intrinsic value was assessed using three items with a sample item being “German is important to me irrespective of the grades”, based on items from a German large-scale study on students’ emotional experiences (Pekrun et al., 2004). Reliability of the scale was good for all subjects (Cronbach’s alpha/omega = 0.70–0.86/0.72–0.87).

##### Self-Concept

Academic self-concept refers to students’ overall academic self-perception in the academic arena. It includes their belief in their own abilities in a specific domain and as such has been found highly influential regarding effort and engagement (e.g., Schnitzler et al., 2021), learning and achievement (e.g., Marsh & Martin, 2011), or career and life choices (e.g., Jansen et al., 2015). The present study assessed ability-related self-concept using three items from the Self-description Questionnaire (Marsh, 1990). A sample item is “I get good marks in German.” Reliability of the scale was good for all subjects (Cronbach’s alpha/omega = 0.86–0.90/0.87–0.90).

##### Career Aspiration

Students’ career orientation in the present study was conceived as the individual aspiration to work in or avoid a field that requires knowledge and skills in the respective subject domain, for instance in the French language. The items were based on selected items from the TIMSS 2011 survey (Mullis et al., 2012) and the 2006 PISA student questionnaire (OECD, 2009) and adapted for the purpose of the present study. The scales for approach and avoidance career orientation were piloted in advance of the present study to check for understanding of item wordings by students and overall consistency and factorial structure of the scale. Sample items are “I would like to have a job in which I get to use my French language skills” for approach career orientation related to French; and “I don’t want to have anything to do with French in the future.” for avoidance career orientation related to French. Reliability of the approach (three items, Cronbach’s alpha/omega = 0.78–0.86/0.85–0.90) as well as the avoidance career orientation (three items for German and Mathematics, four items for English and French, Cronbach’s alpha/omega = 0.84–0.88/0.85–0.88) were good with the exception of German career avoidance which showed acceptable reliability (Cronbach’s alpha/omega = 0.60/0.61).

### Analysis Strategy

Calculations were performed in R 4.3.3 (R Core Team, 2024), using the package mice 3.16.0 for the imputation analyses (van Buuren & Groothuis-Oudshoorn, 2011).

One underlying assumption in the Control Value Theory regarding students’ emotional experiences is the so-called universality assumption (e.g., Goetz et al., 2020). Meaning that while students’ emotions are organized according to academic domains (i.e., their experience of anxiety might be different in mathematics than in German lessons), structural relations are domain-independent (for example, control appraisals are negatively related to anxiety irrespective of the domain). Therefore, to gauge similarity of aggregated states and self-reported traits, we chose to forego discriminating between the four subjects (results herefor are presented in the Supplementary Materials) based on the principle of parsimony.

**Fig. 2:**
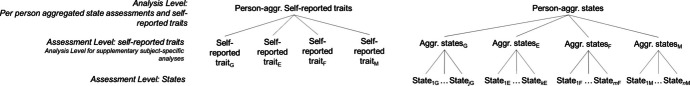
Assessment and analysis level for traits and states, depicted exemplary for one emotion and one wave. G = German, E = English, F = French, and M = mathematics

Analyses were performed on the person-level. In each wave and for each emotion, states were assessed in each subject for a number of lessons, resulting in several state assessments per person. Traits were assessed one-time per suject, with these four assessmentst then also aggregated per person. Assessment and analysis levels for traits, states, and aggregated states are illustrated in Fig. 2.

For investigating convergence (Hypothesis 1), bivariate correlations of aggregated states with self-reported traits were calculated on the person-level for each emotion and each wave T1–T3. We also calculated the average correlation of aggregated states and self-reported traits for each emotions across all three timepoints by Fisher-*z*-transforming the correlations, averaging, and then re-transforming.

Mean-level differences (Hypothesis 2) between the aggregated states and self-reported traits were analyzed by one-sample *t*-tests conducted for each emotion at each timepoint.

Long-term stability of aggregated state and self-reported traits of emotions (Hypothesis 3) were analyzed using autocorrelations. We differentiated between stability over one year (from T1 to T2 and from T2 to T3) and over two years (from T1 to T3). The two 1-year autocorrelations for aggregated states and the two for self-reported traits per emotion were Fisher-*z*-transformed, averaged, and then re-transformed. For each emotion, this procedure resulted in one pair of autocorrelations (i.e., one autocorrelation for aggregated states and one for traits) for the 1-year span and one pair for the 2-year span. The difference between aggregated state- and self-reported trait-autocorrelations was calculated for each pair (Silver et al., 2004) to gauge differences in long-term stability.

Predictive power of aggregated states vs. self-reported traits (Hypothesis 4) was analyzed via semi-partial correlations to determine incremental predictive validity of aggregated states and self-reported traits, respectively. The aggregated state emotions, controlled for their corresponding self-reported traits, were correlated with the outcome criteria. Conversely, self-reported traits of emotions corrected by their corresponding aggregated state emotion were correlated to the outcome criteria. For each emotion, semi-partial correlations were Fisher-*z*-transformed, averaged across waves, and then re-transformed. This resulted in a set of five semi-partial correlations for aggregated state emotions (one per outcome), and five semi-partial correlations for self-reported traits (one per outcome).

## Results

Tables 1 (state) and 2 (self-reported traits) show descriptive statistics of study variables, that is aggregated state emotions and self-reported traits emotions as well as external criteria (domain-specific interest and self-concept, approach-related, and avoidance-related career orientation). Descriptive statistics of study variables according to domains and waves can be found in the Supplementary Materials (Tables S1 and S2). Notably and comparable to previous studies (Ahmed et al., 2010; Moeller et al., 2020; Nett et al., 2017), the majority of variance in students’ momentary emotional experience was on the within-person level as indicated by ICC(1)s ranging between 0.195 (anger at T3) and 0.443 (pride at T3) (*Md*_ICC1_ = 0.255). Based on ICC(2)s being indicative of reliability of aggregate momentary measures (Lüdtke et al., 2006), findings suggest that using person-aggregated emotional states was fairly reliable, i.e., larger than 0.70, with ICC(2)s ranging from 0.735 (anger at T3) to 0.901 (pride at T3) (*Md*_ICC2_ = 0.82).^2^

**Table 1:** Descriptive statistics of state study variables at the three measurement points T1, T2, and T3

	T1						T2						T3					
	*n*	*M*	*SD*_*W*_	*SD*_*B*_	ICC(1)	ICC(2)	*n*	*M*	*SD*_*W*_	*SD*_*B*_	ICC(1)	ICC(2)	*n*	*M*	*SD*_*W*_	*SD*_*B*_	ICC(1)	ICC(2)
Enjoyment	2668	2.85	1.05	0.56	0.222	0.836	1543	2.76	1.02	0.54	0.218	0.770	1258	2.62	0.94	0.57	0.271	0.810
Anger	2666	1.86	1.05	0.53	0.202	0.819	1543	1.95	1.02	0.52	0.197	0.748	1258	1.82	0.94	0.48	0.195	0.735
Pride	2667	2.21	1.05	0.71	0.335	0.900	1543	2.08	1.06	0.65	0.318	0.849	1257	2.03	0.98	0.73	0.443	0.901
Anxiety	2661	1.45	1.00	0.48	0.265	0.866	1543	1.55	0.95	0.46	0.202	0.753	1257	1.44	0.82	0.49	0.286	0.821
Shame	2668	1.36	0.80	0.39	0.231	0.843	1543	1.48	0.92	0.43	0.233	0.786	1257	1.34	0.77	0.41	0.304	0.833
Boredom	2663	2.61	0.70	0.70	0.291	0.880	1543	2.51	0.78	0.61	0.245	0.796	1257	2.46	0.62	0.64	0.278	0.815

All state emotions were rated from (1) *strongly disagree* to (5) *strongly agree*

*n* number of measurements per emotion, aggregated across students and domains (on average, 22 measurements per person); *SD*_*W*_ Standard deviation within students; *SD*_*B*_ Standard deviation between students; *ICC(1)* intraclass correlation coefficient 1 (proportion of between person variance to the total variance); *ICC(2)* intraclass correlation coefficient 2 (reliability of aggregated variable)

**Table 2:** Descriptive statistics of trait study variables for the measurement points T1, T2, and T3

	T1		T2		T3	
	*M*	*SD*	*M*	*SD*	*M*	*SD*
*Trait emotions*						
Enjoyment	2.86	0.71	2.76	0.86	2.67	0.74
Anger	2.14	0.76	2.14	0.79	2.35	0.79
Pride	2.40	0.85	2.50	0.93	2.47	0.83
Anxiety	1.39	0.52	1.57	0.74	1.63	0.69
Shame	1.42	0.54	1.61	0.70	1.66	0.65
Boredom	2.64	0.73	2.76	0.82	3.01	0.76
*Trait external criteria*						
Intrinsic value	3.24	0.54	3.19	0.55	3.07	0.55
Self-concept	3.28	0.64	3.18	0.61	3.10	0.60
Career orientation: approach					2.87	0.50
Career orientation: avoidance					2.51	0.58

*N* = 161 students based on 100 multiply imputed data. All trait items were rated between (1) *not at all* to (5) *very strongly*. External criteria were rate between (1) *strongly disagree* to (5) *strongly agree*. *n* = number of students. Career orientation was only assessed at measurement point T3

### Convergence of Aggregated States and Traits (Hypothesis 1)

In order to answer the first research question regarding the convergence between aggregated state and self-reported traits, aggregated states were correlated with their respective self-reported trait for each emotion and each wave (see Table 3). The across-waves averaged correlations between aggregated states and self-reported traits for enjoyment were *r* = 0.424, for anger *r* = 0.488, for pride *r* = 0.520, for anxiety *r* = 0.535, for shame *r* = 0.391, and for boredom *r* = 0.608. In terms of effect size, the lowest convergence between aggregated states and self-reported traits was observed for shame, for which in fact many of the individual correlations were small (also see Table S3 in the Supplementary Materials). Boredom on the other hand showed the largest relation between aggregated states and self-reported traits. Overall and as expected, aggregated states correlated substantially albeit of varying size with their corresponding self-reported traits.

**Table 3:** Convergence: bivariate correlations of aggregated state with respective trait emotions for the three measurement points T1, T2, and T3

	Correlations of aggregated states with their respective traits			Average correlation across waves
	T1	T2	T3	
Enjoyment	0.413 [0.269, 0.539]	0.509 [0.359, 0.633]	0.344 [0.166, 0.500]	0.424 [0.267, 0.560]
Anger	0.413 [0.270, 0.538]	0.509 [0.362, 0.631]	0.538 [0.382, 0.665]	0.488 [0.339, 0.614]
Pride	0.499 [0.364, 0.613]	0.524 [0.382, 0.642]	0.535 [0.370, 0.668]	0.520 [0.372, 0.642]
Anxiety	0.449 [0.311, 0.368]	0.572 [0.428, 0.687]	0.577 [0.419, 0.700]	0.535 [0.388, 0.656]
Shame	0.217 [0.059, 0.365]	0.487 [0.320, 0.624]	0.451 [0.282, 0.592]	0.391 [0.223, 0.536]
Boredom	0.599 [0.485, 0.693]	0.582 [0.449, 0.689]	0.641 [0.503, 0.747]	0.608 [0.480, 0.710]

*N* = 161 students based on 100 multiply imputed data; all correlations are statistically significant at *p* = 0.05. For the average correlation across waves, the correlations at each wave were Fisher-*z*-transformed, averaged, and then re-transformed. Values in brackets indicate 95% confidence intervals

### Mean-Level Differences of Aggregated States and Traits (Hypothesis 2)

With regard to the second research question on mean-level differences of aggregated states compared to self-reported traits, pairwise *t*-tests were conducted for each emotion (Table 4). Descriptively and with the exception of anxiety, all mean level differences were in the expected direction (with self-reported traits rated higher than aggregated states), although the differences were sometimes small and mostly did not reach statistical significance. Specifically, self-reported traits were rated statistically significantly higher for anger (at T1 and T3), for pride (at all timepoints), for shame (at T3), and for boredom (at T2 and T3). For anxiety, aggregated states were descriptively rated higher than self-reported traits, but the differences were not statistically significant.

**Table 4:** Mean-level differences between aggregated state and trait emotions

	T1						T2						T3					
	*M (SD)*		Difference test trait—state				*M (SD)*		Difference test trait—state				*M (SD)*		Difference test trait—state			
	Agg. state	Trait	*t*	*df*	*p*	*d*	Agg. state	Trait	*t*	*df*	*p*	*d*	Agg. state	Trait	*t*	*df*	*p*	*d*
Enjoyment	2.85 (0.63)	2.86 (0.71)	0.18	138.62	0.855	0.02	2.74 (0.66)	2.76 (0.86)	0.25	95.47	0.801	0.02	2.55 (0.71)	2.67 (0.74)	1.30	72.06	0.199	0.14
Anger	1.86 (0.62)	2.14 (0.76)	4.51	142.83	** < 0.001**	0.37	2.10	2.14 (0.49)	0.54	107.10	0.594	0.05	1.96 (0.71)	2.35 (0.79)	5.22	78.98	** < 0.001**	0.54
Pride	2.19 (0.75)	2.40 (0.85)	3.13	140.34	**0.002**	0.26	2.14 (0.74)	2.50 (0.93)	4.67	108.20	** < 0.001**	0.43	2.12 (0.77)	2.47 (0.83)	4.24	76.55	** < 0.001**	0.44
Anxiety	1.46 (0.54)	1.39 (0.52)	− 1.61	149.56	0.110	− 0.13	1.68 (0.64)	1.57 (0.74)	− 1.84	101.53	0.068	− 0.17	1.62 (0.63)	1.63 (0.69)	0.14	88.51	0.891	0.01
Shame	1.36 (0.41)	1.42 (0.54)	1.15	148.11	0.253	0.09	1.59 (0.56)	1.61 (0.70)	0.24	96.12	0.808	0.02	1.51 (0.56)	1.66 (0.65)	2.33	89.90	**0.022**	0.23
Boredom	2.59 (0.75)	2.64 (0.73)	1.01	139.30	0.314	0.08	2.60 (0.77)	2.76 (0.82)	2.33	110.24	**0.022**	0.21	2.62 (0.82)	3.01 (0.76)	5.46	75.27	**0.001**	0.58

*N* = 161 students based on 100 multiply imputed data; *p*-values in bold were statistically significant at < 0.05. *d* indicates the effect size Cohen’s *d*. *d*-values

### Long-Term Stability of Aggregated States and Traits (Hypothesis 3)

Regarding the long-term stability of aggregated states versus self-reported traits, we calculated autocorrelations across one-year spans (autocorrelations between waves 1 and 2, and between waves 2 and 3) and a two-year span (autocorrelations between waves 1 and 3). The auto correlations are displayed in Table 5 (autocorrelations according to domains and waves are given in the Supplementary Materials, Table S4). Autocorrelations for aggregated states (ranging from 0.408 to 0.561 for the 1-year spans, and from 0.332 to 0.452 for the two-year span) were compared to those of self-reported traits (ranging from 0.493 to 0.590 for 1-year and 0.411 to 0.499 for 2-year spans) and overall found to not significantly differ. Thereby and although descriptively the autocorrelations for aggregated states appear overall smaller than those of self-reported traits for one- and two-year spans, our data revealed no significant difference between the two.

**Table 5:** Long-term stability: average auto-correlations of aggregated states and traits for 1- and 2-year spans

	Average^a^ autocorrelations *r*					
	1-year span (T1–T2; T2–T3)		Pairwise comparison aggregated states—traits	2-year span (T1–T3)		Pairwise comparison aggregated states—traits
	Aggregated states	Traits	*p*	Aggregated states	Traits	*p*
Enjoyment	0.491 [0.335, 0.621]	0.506 [0.350, 0.635]	0.846	0.389 [0.207, 0.544]	0.384 [0.212, 0.533]	0.960
Anger	0.483 [0.330, 0.610]	0.543 [0.395, 0.664]	0.416	0.337 [0.169, 0.486]	0.411 [0.254, 0.547]	0.394
Pride	0.554 [0.411, 0.671]	0.560 [0.409, 0.681]	0.935	0.452 [0.278, 0.597]	0.440 [0.270, 0.583]	0.884
Anxiety	0.505 [0.352, 0.633]	0.552 [0.383, 0.685]	0.512	0.332 [0.164, 0.482]	0.441 [0.270, 0.584]	0.197
Shame	0.408 [0.249, 0.546]	0.493 [0.320, 0.634]	0.309	0.357 [0.196, 0.499]	0.485 [0.321, 0.621]	0.148
Boredom	0.561 [0.418, 0.677]	0.590 [0.453, 0.700]	0.650	0.426 [0.257, 0.569]	0.499 [0.341, 0.631]	0.311

*N* = 161 students based on 100 multiply imputed data

^a^Aggregated states and traits were averaged for the two 1-year spans T1–T2 and T2–T3. Values in brackets indicate 95% confidence intervals

### Predictive Power of Aggregated States and Traits (Hypothesis 4)

Regarding predictive power of aggregated states vs. self-reported traits, we calculated semi-partial correlations of aggregated states controlling for self-reported traits (and vice versa) with external criteria of grades, intrinsic value, self-concept and career orientation. Across-wave averaged semi-partial correlations are summarized in Table 6 (semi-partial correlations according to domains and waves are given in the Supplementary Materials Table S5). Overall, self-reported traits (corrected for aggregated emotional states) were only systematically correlated to external criteria for students’ enjoyment and somewhat for pride. Further, self-reported traits of anger corrected for aggregated state anger was positively correlated to avoidance career orientation, and self-reported traits of boredom corrected for aggregated state boredom was negatively correlated to intrinsic value. There was only one significant correlation with external criteria of aggregated state emotions when correcting for self-reported traits and that was for enjoyment being negatively related to avoidance career orientation. This lends to the conclusion that for some but not all emotions self-reported traits corrected for aggregated states contribute something to the prediction of external criteria, whereas the opposite does not seem to be the case.

**Table 6:** Incremental predictive validity: semi-partial correlations of aggregated states and traits with grade, intrinsic value, self-concept and (approach, avoidance) career orientations

		Grade	Intrinsic value	Self-concept	Career orientation	
					Approach	Avoidance
Enjoyment	Traits	**0.269** [0.101, 0.436]	**0.599** [0.460, 0.739]	**0.283** [0.220, 0.538]	**0.352** [0.203, 0.502]	− **0.269** [− 0.420, − 0.119]
	Agg. states	0.026 [− 0.141, 0.193]	0.006 [− 0.134, 0.145]	0.111 [− 0.103, 0.216]	0.179 [0.029, 0.328]	− **0.253** [− 0.404, − 0.102]
Anger	Traits	− 0.157 [− 0.355, 0.020]	− 0.145 [− 0.323, 0.032]	− 0.182 [− 0.359, − 0.005]	− 0.062 [− 0.246, 0.122]	**0.261** [0.086, 0.437]
	Agg. states	− 0.025 [− 0.203, 0.162]	− 0.041 [− 0.219, 0.138]	0.006 [− 0.173, 0.185]	− 0.108 [− 0.292, 0.076]	0.110 [− 0.066, 0.285]
Pride	Traits	**0.184** [0.002, 0.365]	**0.369** [0.198, 0.539]	**0.277** [0.103, 0.452]	0.105 [− 0.076, 0.287]	− 0.084 [− 0.267, 0.099]
	Agg. states	− 0.014 [− 0.195, 0.167]	0.024 [− 0.146, 0.193]	0.069 [− 0.106, 0.243]	0.136 [− 0.045, 0.318]	− 0.108 [− 0.291, 0.076]
Anxiety	Traits	− 0.110 [− 0.296, 0.076]	− 0.051 [− 0.237, 0.135]	− 0.076 [− 0.262, 0.109]	− 0.121 [− 0.311, 0.068]	0.177 [− 0.006, 0.360]
	Agg. states	0.073 [− 0.113, 0.258]	0.095 [− 0.092, 0.281]	0.061 [− 0.127, 0.248]	− 0.047 [− 0.237, 0.143]	0.169 [− 0.014, 0.351]
Shame	Traits	− 0.075 [− 0.247, 0.096]	0.053 [− 0.119, 0.225]	− 0.019 [− 0.190, 0.152]	0.018 [− 0.156, 0.191]	0.182 [0.014, 0.350]
	Agg. states	0.027 [− 0.145, 0.198]	0.030 [− 0.143, 0.202]	0.017 [− 0.157, 0.190]	− 0.166 [− 0.340, 0.007]	0.164 [− 0.004, 0.332]
Boredom	Traits	− 0.161 [− 0.323, 0.063]	− **0.298** [− 0.445, − 0.075]	− 0.153 [− 0.362, 0.025]	− 0.146 [− 0.344, 0.051]	**0.242** [0.056,0.427]
	Agg. states	− 0.043 [− 0.268, 0.118]	− 0.098 [− 0.287, 0.087]	− 0.040 [− 0.175, 0.213]	− 0.141 [− 0.338, 0.057]	**0.224** [0.038, 0.409]

Semi-partial correlations of traits with external criteria were corrected for aggregated states and vice versa. The semi-partial correlations of traits and aggregated states with the external criteria were Fisher-*z*-transformed, averaged across T1, T2, and T3, and then re-transformed. If semi-partial correlations were statistically significant at *p* < 0.05 in at least one wave, they are printed bold in the table. Values in brackets indicate 95% confidence intervals

## Discussion

The present study aimed to compare two trait emotion measures—students’ aggregated emotional states and self-reported traits—to examine the extent of their similarity. Understanding this relationship is crucial for future studies that seek to investigate emotional experiences at a person-level, given that students’ emotions are dynamic and context-dependent.

### Key Findings on Similarity

In terms of convergence (Hypothesis 1), the present study largely corroborated previous findings (Finnigan & Vazire, 2018; Röcke et al., 2011; Wirtz et al., 2003). That is, correlations between aggregated states and self-reported traits (averaged across the four domains and across the three time points) ranged between 0.391 and 0.608. Comparing that to the two previous studies we know of using discrete emotions, the correlation reported for boredom, *r* = 0.50, by Krannich et al. (2022) was within the same effect size range as the one currently found, *r* = 0.60, whereas the correlations reported by Pekrun et al. (2017) were consistently larger. Specifically, Pekrun et al. (2017) reported correlations of *r* = 0.65 for enjoyment (compared to presently found *r* = 0.424), 0.70 for frustration/anger (compared to 0.488), 0.76 for anxiety (0.535), and 0.82 for boredom (0.608). However, the differences in effect sizes can be explained when looking at the specifics of the trait-measure used by Pekrun et al. (2017): in an experimental study participants read four texts and rated their state emotions immediately after reading the first three. However, their trait-like emotion was obtained by asking particpants after having read all four texts to once retrospectively rate their emotions when reading the texts. Thereby, that specific trait-like measure attaches a fixed time-frame (see also Wirtz et al., 2003), whereas the presently used self-reported trait assessed emotions generalized and globally (i.e., emotions “usually” experienced in a specific domain, see item wordings in Table 7). Or in the words of Conner and Barrett (2012), the traits in the Pekrun et al. (2017) study represent the retrospective, remembering self and thus are closer to the actual experience than the presently used traits representing the believing self.

Regarding the second criterion for evaluating similarity, the observed memory experience gap (Hypothesis 2) in the present study is somewhat consistent with previous findings, but with notable differences. That is, the extent of the memory experience gap varied across emotions. Across the three time-points, aggregated states and self-reported traits exhibited mean-level differences for pride, anger, and boredom. Aggregated states and self-reported traits differed for only one timepoint for shame, and for none of the three time-points for anxiety and enjoyment. Thereby, effect-sizes for the statistically significant differences were small to moderate (Cohen’s *d* = 0.21–0.58). In sum, mean-level differences were not as consistent as previous studies suggested; that is, the presently found differences were not always consistent over three consecutive years and contrary to previous findings (Miron-Shatz et al., 2009); our data showed no systematic larger differences in unpleasant compared to pleasant emotions. Also, mean-level differences were not uniform across all discrete emotions.

Assumptions for higher long-term stability of self-reported traits compared to aggregated states were not supported within the present study (Hypothesis 3). That is, while the auto-correlations across 1-year and 2-year time spans were descriptively larger for self-reported traits than for aggregated states, the differences were not statistically significant. This is not to say that self-reported traits and aggregated state emotions were not stable over time. In fact, the presently found auto-correlations were moderate in size, ranging between 0.332 and 0.590. Self-reported traits were not more stable than aggregated states. Given that descriptively auto-correlations of self-reported traits were consistently larger than those of aggregated states, our findings warrant replication to check whether these differences are meaningful or not. This appears particularly imperative, considering that apart from the present study and that of Rauthmann et al. (2018), we are not aware of any other study comparing the long-term stability of aggregated states and self-reported traits.

Finally and regarding incremental predictive validity (Hypothesis 4), we found that self-reported traits predicted the outcomes (grades, intrinsic value, self-concept, and career orientation) in the expected direction over and above aggregated states for enjoyment and pride, but not or only unsystematically for anger, anxiety, shame and boredom. Conversely, aggregated states over and above self-reported traits did not systematically predict outcomes across emotions. Our findings complement a longitudinal study on students’ emotional traits (Pekrun et al., 2017) that examined enjoyment, anger, pride, anxiety, and shame and found them to consistently relate to achievement. In sum, our findings support conceptual considerations that traits unlike states, incoporate identity-related knowledge and should thus be more predicitve of outcomes than states (Conner & Barrett, 2012; Robinson & Clore, 2002), but also that this assumption does not apply uniformly to all emotions.

### Theoretical and Practical Implications

It is widely accepted that states and traits capture different aspects of emotional experiences. Self-reported traits, such as the one used in the current study, are commonly employed to measure emotional traits defined as a predisposition to react to certain situations in a consistent way (e.g., Endler & Kocovski, 2001). Trait theories (e.g., Epstein, 1979) and their methodological counterparts, such as latent state trait theory (Geiser et al., 2017), suggest that these momentary reactions (i.e. states) can be sampled and, if they cover a wide range of diverse situations, aggregated to approximate traits. The present study found that these two approaches—using self-reported traits and aggregating states—are similar in some ways, such as in terms of convergence and long-term stability, but differ in others, particularly in mean-level differences and their predictive power for outcomes. The extent of similarity also varies depending on the discrete emotion being assessed. These differences highlight the need for further investigation into how students’ momentary emotional experiences accumulate across various contexts and over time to shape or inform their knowledge about their emotional experiences.

From a practical standpoint, our findings indicate that general recommendations favoring one measure over the other—whether self-reported traits or aggregating states—likely require a more nuanced and detailed approach. Depending on the specific research objectives and theoretical frameworks, the selection of either aggregated states or self-reported traits should be deliberate and purposeful. Our study underscores the importance of these considerations and provides insights meant to guide researchers in making informed decisions.

### Limitations

The present study’s limitations primarily arise from the chosen assessments. State assessments of students’ discrete emotions were based on single items (i.e., one item per emotion). This choice was based on the one hand on previous studies using similar approaches (e.g., Ketonen et al., 2018) and findings that suggest validity of single items for affective constructs (e.g., Gogol et al., 2014). On the other hand, multi-item scales and hence the longer time it takes to complete them compared to single-item assessments might either disrupt classroom processes or interfere with the assessment of the student’s emotional state itself by drawing out the time since the cue to report sounded and thus making access to emotion information stored within the episodic memory more difficult (see Robinson & Clore, 2002). The decision to forego multi-item scales in favor of capturing a broader spectrum across discrete emotions doesn't necessarily dictate the approach for future studies. These studies could focus on fewer emotions, but assess them in more detail with a larger number of items than is currently the case. This approach would ameliorate a second limitation, namely the reliability of per-person aggregated self-reported traits. Due to the single-item assessment of the self-reported traits, our measure on the person-level on which we did all analyses (for domain-specific results, see Supplementary Materials) may have had a low reliability or at least lower reliability than the per-person aggregated states, resulting in an attenuation of the observed correlations, for example that between self-reported traits and aggregated states.

Another issue could stem from the item wording in conjunction with the response scale used in the assessment of states vs. self-reported traits. Specifically, state emotions were assessed with the item “At the moment, I am happy” (for enjoyment), using a five-point response-scale ranging from (1) *not at all* to (5) *very strongly*. Therefore, the state emotion assessment measures the strength of momentary joy. Aggregated state emotions, in turn, reflect how frequently the emotion was experienced to some extent over a ten-day period. Or, in other words, the evaluation of strength and frequency is mixed in the aggregated states. In contrast, self-reported traits were assessed with the item “In English lessons, I am usually happy” (for enjoyment in English), using the same response-scale as for the state assessments. Thus, self-reported traits measure the frequency of experiencing an emotion in class. While comparing the mean levels of aggregated states with self-reported traits is statistically plausible (due to the parallel item wording and identical response scale), it raises the question of whether we are actually comparing different aspects—strength and frequency—of emotional experiences. Future studies should explore this issue and, for example, clarify whether students place more importance on strength or frequency when answering items such as ours, potentially through cognitive item validation procedures.

Also, the data this study is based on did only include a limited range of outcome criteria with which to investigate incremental predictive validity. While grades are an achievement outcome, self-concept, intrinsic value, and to some extent also career aspirations are motivational outcomes. Aggregated state emotions may be more important compared to trait emotions when determining an individual’s overall well-being as the experiencing self should be more strongly connected to bodily processes than the remembering self (Conner & Barrett, 2012). Future studies therefore need to challenge our finding of aggregated states evidencing no incremental predictive validity to self-reported traits with regard to outcomes more closely tied to (bodily) well-being. Furthermore, external criteria were assessed simultaneously with self-reported traits, which may have led to an overestimation of the relationship between self-reported traits and outcomes, compared to aggregated states. However, despite this potential bias, the incremental predictive power of self-reported traits, controlling for aggregated states, was only systematically observed for enjoyment and, to a lesser extent, pride. This suggests that the method bias likely did not significantly impact the current findings.

The findings of the present study, particularly regarding predictive validity, are limited by the asymmetry in time frames between states and self-reported traits. State emotions were assessed over a ten-day period during English, German, French, and Mathematics classes with items such as “At the moment, I am happy,” while the corresponding self-reported trait item was “I am usually happy,” referring to one of the four domains. Thus, while both states and self-reported traits covered the same contexts, they did not align in terms of time frames. As posited by Robinson and Clore (2002), momentary emotional self-reports rely on episodic memory. However, as the recall period increases, as in self-reported traits, the emotional events become less immediately accessible in memory. Instead, individuals draw on semantic knowledge influenced by their beliefs about themselves and their experiences. Given that our outcome criteria for the fourth research question on incremental predictive validity—except for grades—are also reflective of ability-, value-, and career-related beliefs, this may distort our predictions in favor of self-reported traits of aggregated states.

Differing time frames in state and self-reported traits of emotions could also have impacted the observed memory-experience gap. To cover the same range of emotional experiences as self-reported traits, state assessments would need to span all possible emotion-eliciting events. This was arguably not the case in the present study, where the ESM phase occurred during regular classes, thus capturing a range of learning activities and social interactions, but specifically excluding exams. Previous research (e.g., Spangler et al., 2002) shows that exams, including preparation and performance feedback, are highly emotionally charged events for students. As evidenced by the research on test anxiety, students experience a range of emotions, including high activation ones like hope, pride, and anger (Pekrun et al., 2004; Schutz et al., 2008). These peak emotional experiences would have been captured in the current global trait self-reports, but not in the aggregated states. As a result, the means of self-reported traits would likely be higher compared to aggregated states. This may help explain the systematic mean-level differences observed for anger and pride. However, no such systematic differences were found for anxiety, suggesting that the asymmetry in emotional event coverage between states and self-reported traits might not have significantly affected our findings.

In additional analyses, we examined how extending time frames and increasing the coverage of emotional events—by aggregating states across all waves—affected their correlation to (wave-specific and across waves) self-reported traits. The resulting correlations (see Supplementary Table S6) were largely similar to those obtained when states were aggregated per wave (see Table 3). Nevertheless, future studies comparing aggregated states to self-reported traits should aim to achieve greater symmetry in the assessments, particularly regarding time frames.

## Conclusion

The major finding of the present study is that there is no significant benefit or harm in using aggregated states instead of self-reported traits when investigating students’ emotional lives on a person-level. However, it is essential to consider the trade-offs involved: while aggregated states can serve as a viable alternative for self-reported traits, the additional burden of gathering multiple states instead of self-reported traits may not always be justifiable, particularly in terms of research resource allocation and efficiency with regard to students’ testing time. Moreover, it is imperative to recognize that despite the observed convergence and similar stability between aggregated states and self-reported traits, there were also some differences, particularly with regard to mean-level differences. In conclusion, our study contributes to the ongoing discourse surrounding the utilization of states and traits in investigating (students’) emotional experiences. By evidencing (dis)similarities between aggregated states and self-reported traits based on several indicators, we aim to inform and guide researchers in making methodological choices that align with the goals and objectives of their studies.

## Electronic supplementary material

Below is the link to the electronic supplementary material.

Supplementary file1 (DOCX 101 KB)

## Data Availability

The data for the paper is available upon request from the corresponding author.

## Appendix

See Table 7.

**Table 7:** Item wordings for students’ emotions

Student emotion	Item wording	
	Trait	State
	How are you usually in DOMAIN classes? *Wie geht es Dir im [Fach]unterricht in der Regel?*	
Enjoyment	In DOMAIN classes, I am usually happy *Im FACHunterricht freue ich mich in der Regel*	At the moment, I am happy *Im Moment freue ich mich*
Anger	In DOMAIN classes, I am usually angry *Im FACHunterricht ärgere ich mich in der Regel*	At the moment, I am angry *Im Moment ärgere ich mich*
Pride	In DOMAIN classes, I am usually proud *Im FACHunterricht bin ich in der Regel stolz*	At the moment, I am proud *Im Moment bin ich stolz*
Anxiety	In DOMAIN classes, I am usually anxious *Im FACHunterricht habe ich in der Regel Angst*	At the moment, I am anxious *Im Moment habe ich Angst*
Shame	In DOMAIN classes, I am usually ashamed *Im FACHunterricht schäme ich mich in der Regel*	At the moment, I am ashamed *Im Moment schäme ich mich*
Boredom	In DOMAIN classes, I am usually bored *Im FACHunterricht langweile ich mich in der Regel*	At the moment, I am bored *Im Moment langweile ich mich*

Item wordings give the original German wording (in italics) as well as their translation to English. Self-reported trait emotions and state emotions were rated on a five-point scale from (1) *not at all* to (5) *very strongly*

See Table 8.

**Table 8:** Item wordings for students’ grades, intrinsic value, self-concept, and career orientation

Item	Item wording
Grade	Which grade did you receive in DOMAIN in your last school report? *Welche Note hattest du auf dem letzten Zeugnis in DOMAIN?*
Intrinsic value	DOMAIN is important to me regardless of the grades *FACH ist mir unabhängig von der Note sehr wichtig*
	I consider DOMAIN to be very important *Ich halte das Fach Deutsch für sehr wichtig*
	DOMAIN is my favorite academic subject *Deutsch ist mein Lieblingsfach*
Self-concept	I get good marks in DOMAIN *Im FACH bekomme ich gute Noten*
	DOMAIN is one of my best domains *FACH ist eines meiner besten Fächer*
	I have always done well in DOMAIN *Ich war schon immer gut in FACH*
Career orientation: approach	
French & English:	I would like to have a job in which I get to use my French/English language skills *Ich würde später gerne in einem Beruf arbeiten, in dem ich meine Französisch-/Englischkenntnisse intensiv nutzen kann*
	I would like to work for an organization where I can be in contact with French/English speaking countries and people *Ich würde später gerne bei einer Organisation arbeiten, in der ich in Kontakt mit französisch-/englischsprachigen Ländern bzw. Menschen stehen kann*
	I could image doing something in my future career that requires a lot of French/English *Ich könnte mir vorstellen in meiner beruflichen Zukunft etwas zu machen, bei dem man viel Französisch/Englisch braucht*
Mathematics:	I can image having a job that requires a lot of math skills *Ich kann mir vorstellen in der Zukunft etwas zu machen, bei dem man viel Mathe braucht*
	I am considering a career in mathematics, physics or engineering *Ich ziehe in Erwägung, einen Beruf im mathematischen, physikalischen oder technischen Bereich zu ergreifen*
	I would like to work in a field in which my mathematical skills play a role *Ich möchte später einmal in einem Bereich arbeiten, in dem meine mathematischen Fähigkeiten eine Rolle spielen*
German:	I would like to work in a profession that requires me to have good German grammar and spelling skills *Ich würde später gerne in einem Beruf arbeiten, der voraussetzt, dass ich über eine gute deutsche Grammatik und Rechtschreibung verfüge*
	I could imagine working in a profession where my German language skills are particularly in demand *Ich könnte mir vorstellen einen Beruf zu ergreifen, in dem meine deutschsprachigen Kompetenzen besonders gefragt sind*
	I would be happy if it is important in my future job to have good oral and written language skills in German *Ich würde mich freuen, wenn es in meinem späteren Beruf wichtig ist, gute mündliche und schriftliche Sprachkompetenzen in Deutsch zu haben*
Career orientation: avoidance	
French & English:	If possible, I don’t want to do a job later on where I have to speak French/English *Ich möchte später möglichst keinen Job machen, in dem man Französisch/Englisch sprechen muss*
	I don’t want to have anything to do with French/as little to do with English as possible in the future *Ich möchte in Zukunft nichts mehr mit Französisch/möglichst wenig mit Englisch zu tun haben*
	I would be put off if a job advertisement asked for a good knowledge of French/English *Es würde mich abschrecken, wenn bei einer Stellenausschreibung gute Französisch-/Englischkenntnisse gefordert sind*
	I would like to avoid doing anything to do with French/English in my future career *Ich möchte es in meiner beruflichen Zukunft möglichst vermeiden, etwas zu machen, das mit Französisch/Englisch zu tun hat*
Mathematics:	I would rather not have a job that would require strong math skills *Ich möchte später möglichst keinen Job machen, in dem man gute Mathematikkompetenzen benötigt*
	I don’t want to have anything to do with math in my professional future *Ich möchte in meiner beruflichen Zukunft nichts mehr mit Mathematik zu tun haben*
	I would be put off if a job advertisement asked for good math skills *Es würde mich abschrecken, wenn bei einer Stellenausschreibung gute Mathematikkenntnisse gefordert sind*
German:	If possible, I don’t want to do a job later on where you have to be able to express yourself particularly well in German *Ich möchte später möglichst keinen Job machen, in dem man sich in der deutschen Sprache besonders gut ausdrücken können muss*
	I would be put off if a job advertisement demanded very good German language skills (expression, spelling, grammar) *Es würde mich abschrecken, wenn bei einer Stellenausschreibung sehr gute Deutschkenntnisse (Ausdruck, Rechtschreibung, Grammatik) gefordert werden*
	In my professional future, I would like to have as little as possible to do with things that we did in German lessons (literature analysis, grammar, essays, etc.) *Ich möchte in meiner beruflichen Zukunft möglichst wenig mit Sachen zu tun haben, die wir im Deutschunterricht gemacht haben (Literaturanalysen, Grammatik, Aufsätze, usw.)*

Items for the external criteria were assessed in Wave 3 at the post-test, i.e. after the last experience sampling phase. Item wordings give the original German wording (in italics) as well as their translation to English. All items were rated from (1) *strongly disagree* to (5) *strongly agree*

## Footnotes

1 This position is encapsulated in another foundational work, namely the famous equation of Lewin (1951), *B* = *f* (*P*, *S*), expressing that (momentary) behavior *B* is a function of the person *P* and the situation *S*.

2 The ICC(2) is calculated by applying the applying the Spearman-Brown formula to the ICC(1) with the number of measurements replacing the number of items. Thus, the reliability of the aggregated state emotions is a function of the ICC(1), and the number of measurements.
