# Supplementary material for: How Similar Are Students’ Aggregated State Emotions to Their Self-Reported Trait Emotions? Results from a Measurement Burst Design Across Three School Years
Source: Educ Psychol Rev. 2025 Mar 13;37(1):26. doi: 10.1007/s10648-025-09995-1 (PMC11906532; doi:10.1007/s10648-025-09995-1)
Supplement: Supplementary file 1 — Supplementary file1 (DOCX 101 KB) [file 10648_2025_9995_MOESM1_ESM.docx]

How similar are students’ aggregated state emotions to their self-reported trait emotions? Results from a measurement burst design across three school years

Supplementary Materials

# Supplementary Materials

Table S1

*Descriptive Statistics of Aggregated State Emotions According to Waves and Subject Domains*

|  | T1 | | | | | |  | T2 | | | | | |  | T3 | | | | | |
| --- | --- | --- | --- | --- | --- | --- | --- | --- | --- | --- | --- | --- | --- | --- | --- | --- | --- | --- | --- | --- |
|  | *n* | *M* | *SD*_W_ | *SD*_B_ | ICC(1) | ICC(2) |  | *n* | *M* | *SD*_W_ | *SD*_B_ | ICC(1) | ICC(2) |  | *n* | *M* | *SD*_W_ | *SD*_B_ | ICC(1) | ICC(2) |
| Enjoyment |  |  |  |  |  |  |  |  |  |  |  |  |  |  |  |  |  |  |  |  |
| German | 140 | 2.92 | 0.97 | 0.63 | 0.302 | 0.657 |  | 112 | 2.76 | 0.98 | 0.60 | 0.282 | 0.508 |  | 98 | 2.78 | 0.94 | 0.51 | 0.235 | 0.456 |
| English | 138 | 2.95 | 1.04 | 0.55 | 0.229 | 0.533 |  | 115 | 2.92 | 0.94 | 0.61 | 0.308 | 0.565 |  | 95 | 2.79 | 0.85 | 0.65 | 0.384 | 0.620 |
| French | 144 | 2.82 | 0.98 | 0.67 | 0.321 | 0.669 |  | 103 | 2.78 | 1.00 | 0.66 | 0.344 | 0.600 |  | 92 | 2.41 | 0.92 | 0.67 | 0.382 | 0.636 |
| Mathematics | 140 | 2.74 | 0.99 | 0.67 | 0.322 | 0.695 |  | 116 | 2.65 | 0.97 | 0.65 | 0.339 | 0.657 |  | 99 | 2.51 | 0.86 | 0.65 | 0.377 | 0.670 |
| Anger |  |  |  |  |  |  |  |  |  |  |  |  |  |  |  |  |  |  |  |  |
| German | 140 | 1.71 | 0.95 | 0.48 | 0.205 | 0.533 |  | 112 | 1.86 | 0.97 | 0.52 | 0.235 | 0.447 |  | 98 | 1.68 | 0.88 | 0.57 | 0.299 | 0.537 |
| English | 138 | 1.73 | 0.99 | 0.51 | 0.229 | 0.533 |  | 115 | 1.78 | 0.92 | 0.58 | 0.293 | 0.548 |  | 95 | 1.73 | 0.99 | 0.32 | 0.120 | 0.263 |
| French | 144 | 1.83 | 1.02 | 0.46 | 0.173 | 0.472 |  | 103 | 2.02 | 1.10 | 0.56 | 0.256 | 0.496 |  | 92 | 1.95 | 0.88 | 0.68 | 0.416 | 0.668 |
| Mathematics | 140 | 2.08 | 1.03 | 0.74 | 0.347 | 0.719 |  | 116 | 2.15 | 1.04 | 0.72 | 0.330 | 0.647 |  | 99 | 1.79 | 0.93 | 0.60 | 0.321 | 0.614 |
| Pride |  |  |  |  |  |  |  |  |  |  |  |  |  |  |  |  |  |  |  |  |
| German | 140 | 2.20 | 0.91 | 0.76 | 0.426 | 0.767 |  | 112 | 2.04 | 0.92 | 0.63 | 0.344 | 0.580 |  | 98 | 2.09 | 0.88 | 0.72 | 0.414 | 0.657 |
| English | 138 | 2.20 | 1.02 | 0.71 | 0.337 | 0.661 |  | 115 | 2.08 | 0.97 | 0.64 | 0.315 | 0.573 |  | 95 | 1.99 | 0.80 | 0.77 | 0.510 | 0.731 |
| French | 144 | 2.25 | 0.98 | 0.71 | 0.345 | 0.693 |  | 103 | 2.07 | 0.94 | 0.67 | 0.370 | 0.627 |  | 92 | 1.99 | 0.75 | 0.81 | 0.568 | 0.787 |
| Mathematics | 140 | 2.12 | 0.98 | 0.77 | 0.399 | 0.761 |  | 116 | 2.04 | 0.92 | 0.77 | 0.421 | 0.731 |  | 99 | 1.96 | 0.78 | 0.77 | 0.503 | 0.773 |
| Anxiety |  |  |  |  |  |  |  |  |  |  |  |  |  |  |  |  |  |  |  |  |
| German | 140 | 1.39 | 0.69 | 0.48 | 0.336 | 0.691 |  | 112 | 1.45 | 0.84 | 0.39 | 0.176 | 0.360 |  | 98 | 1.35 | 0.76 | 0.36 | 0.206 | 0.413 |
| English | 138 | 1.40 | 0.68 | 0.55 | 0.403 | 0.721 |  | 115 | 1.38 | 0.80 | 0.33 | 0.150 | 0.340 |  | 95 | 1.32 | 0.65 | 0.41 | 0.282 | 0.506 |
| French | 144 | 1.45 | 0.78 | 0.43 | 0.245 | 0.581 |  | 103 | 1.57 | 0.93 | 0.5 | 0.287 | 0.535 |  | 92 | 1.58 | 0.79 | 0.69 | 0.473 | 0.716 |
| Mathematics | 140 | 1.53 | 0.78 | 0.67 | 0.433 | 0.786 |  | 116 | 1.71 | 0.98 | 0.65 | 0.328 | 0.645 |  | 99 | 1.41 | 0.73 | 0.44 | 0.293 | 0.582 |

| Table S1 continued |  | | | | | |  |  | | | | | |  |  | | | | | |
| --- | --- | --- | --- | --- | --- | --- | --- | --- | --- | --- | --- | --- | --- | --- | --- | --- | --- | --- | --- | --- |
|  | T1 | | | | | |  | T2 | | | | | |  | T3 | | | | | |
|  | *n* | *M* | *SD*_W_ | *SD*_B_ | ICC(1) | ICC(2) |  | *n* | *M* | *SD*_W_ | *SD*_B_ | ICC(1) | ICC(2) |  | *n* | *M* | *SD*_W_ | *SD*_B_ | ICC(1) | ICC(2) |
| Shame |  |  |  |  |  |  |  |  |  |  |  |  |  |  |  |  |  |  |  |  |
| German | 140 | 1.31 | 0.60 | 0.39 | 0.312 | 0.668 |  | 112 | 1.34 | 0.65 | 0.42 | 0.302 | 0.532 |  | 98 | 1.29 | 0.51 | 0.46 | 0.448 | 0.688 |
| English | 138 | 1.32 | 0.66 | 0.35 | 0.243 | 0.551 |  | 115 | 1.38 | 0.71 | 0.31 | 0.224 | 0.459 |  | 95 | 1.29 | 0.61 | 0.31 | 0.230 | 0.438 |
| French | 144 | 1.32 | 0.63 | 0.31 | 0.194 | 0.509 |  | 103 | 1.50 | 0.78 | 0.51 | 0.352 | 0.608 |  | 92 | 1.39 | 0.69 | 0.46 | 0.358 | 0.611 |
| Mathematics | 140 | 1.42 | 0.72 | 0.55 | 0.367 | 0.736 |  | 116 | 1.63 | 0.83 | 0.61 | 0.355 | 0.673 |  | 99 | 1.33 | 0.6 | 0.35 | 0.294 | 0.583 |
| Boredom |  |  |  |  |  |  |  |  |  |  |  |  |  |  |  |  |  |  |  |  |
| German | 140 | 2.61 | 0.96 | 0.86 | 0.464 | 0.793 |  | 112 | 2.59 | 1.07 | 0.73 | 0.339 | 0.575 |  | 98 | 2.48 | 1.01 | 0.67 | 0.307 | 0.547 |
| English | 138 | 2.55 | 1.03 | 0.76 | 0.364 | 0.687 |  | 115 | 2.41 | 0.87 | 0.82 | 0.485 | 0.734 |  | 95 | 2.28 | 0.90 | 0.78 | 0.454 | 0.684 |
| French | 144 | 2.62 | 0.94 | 0.88 | 0.478 | 0.797 |  | 103 | 2.48 | 1.05 | 0.65 | 0.306 | 0.558 |  | 92 | 2.64 | 1.01 | 0.76 | 0.397 | 0.650 |
| Mathematics | 140 | 2.56 | 1.04 | 0.75 | 0.347 | 0.718 |  | 116 | 2.49 | 0.96 | 0.76 | 0.408 | 0.72 |  | 99 | 2.41 | 0.87 | 0.78 | 0.474 | 0.752 |

*Note*. *n* = number of students; SD_W_ = Standard deviation within students; SD_B_ = Standard deviation between students; ICC(1) = intraclass correlation coefficient 1 (proportion of between classroom variance to the total variance); ICC(2) = intraclass correlation coefficient 2 (reliability of aggregated variable)

Table S2

*Descriptive Statistics of Trait Emotions and Trait External Criteria According to Waves and Subject Domains*

|  | T1 | |  | T2 | | |  | T3 | |
| --- | --- | --- | --- | --- | --- | --- | --- | --- | --- |
|  | *M* | *SD* |  | *M* | *SD* |  | | *M* | *SD* |
| Enjoyment |  |  |  |  |  |  | |  |  |
| German | 2.86 | 1.04 |  | 2.69 | 1.19 |  | | 2.69 | 1.11 |
| English | 3.16 | 1.18 |  | 3.08 | 1.26 |  | | 2.87 | 1.19 |
| French | 2.84 | 1.12 |  | 2.66 | 1.14 |  | | 2.53 | 1.12 |
| Mathematics | 2.57 | 1.16 |  | 2.60 | 1.22 |  | | 2.58 | 1.08 |
| Anger |  |  |  |  |  |  | |  |  |
| German | 1.93 | 1.05 |  | 2.03 | 1.16 |  | | 2.07 | 1.12 |
| English | 1.97 | 1.12 |  | 1.86 | 1.12 |  | | 2.19 | 1.30 |
| French | 2.03 | 1.08 |  | 2.22 | 1.25 |  | | 2.48 | 1.18 |
| Mathematics | 2.57 | 1.31 |  | 2.44 | 1.23 |  | | 2.67 | 1.27 |
| Pride |  |  |  |  |  |  | |  |  |
| German | 2.31 | 1.09 |  | 2.34 | 1.21 |  | | 2.36 | 1.13 |
| English | 2.65 | 1.21 |  | 2.73 | 1.26 |  | | 2.66 | 1.21 |
| French | 2.34 | 1.12 |  | 2.53 | 1.31 |  | | 2.40 | 1.19 |
| Mathematics | 2.39 | 1.09 |  | 2.40 | 1.29 |  | | 2.44 | 1.23 |
| Anxiety |  |  |  |  |  |  | |  |  |
| German | 1.24 | 0.61 |  | 1.42 | 0.88 |  | | 1.44 | 0.85 |
| English | 1.29 | 0.65 |  | 1.42 | 0.84 |  | | 1.45 | 0.83 |
| French | 1.36 | 0.77 |  | 1.59 | 1.01 |  | | 1.71 | 1.10 |
| Mathematics | 1.66 | 1.04 |  | 1.85 | 1.21 |  | | 1.92 | 1.15 |
| Shame |  |  |  |  |  |  | |  |  |
| German | 1.42 | 0.75 |  | 1.49 | 0.82 |  | | 1.44 | 0.79 |
| English | 1.38 | 0.72 |  | 1.50 | 0.91 |  | | 1.72 | 0.97 |
| French | 1.40 | 0.71 |  | 1.60 | 1.03 |  | | 1.70 | 0.97 |
| Mathematics | 1.47 | 0.85 |  | 1.85 | 1.14 |  | | 1.76 | 1.03 |
| Boredom |  |  |  |  |  |  | |  |  |
| German | 2.80 | 1.16 |  | 2.84 | 1.31 |  | | 3.20 | 1.11 |
| English | 2.45 | 1.15 |  | 2.62 | 1.26 |  | | 2.88 | 1.30 |
| French | 2.71 | 1.14 |  | 2.81 | 1.19 |  | | 3.02 | 1.26 |
| Mathematics | 2.62 | 1.27 |  | 2.77 | 1.21 |  | | 2.92 | 1.19 |

| Table S2 continued | | | | | | | |  |  |  |  |  |  |  |  |  |
| --- | --- | --- | --- | --- | --- | --- | --- | --- | --- | --- | --- | --- | --- | --- | --- | --- |
|  | T1 | |  | | T2 | | | | |  | | T3 | | | | |
|  | *M* | *SD* |  | | *M* | | | *SD* | |  | | *M* | | *SD* | |  |
| Intrinsic Value |  |  |  | |  | | |  | |  | |  | |  | |  |
| German | 3.10 | 0.85 |  | | 3.06 | | | 0.92 | |  | | 2.89 | | 0.90 | |  |
| English | 3.85 | 0.78 |  | | 3.89 | | | 0.71 | |  | | 3.80 | | 0.66 | |  |
| French | 3.03 | 1.04 |  | | 2.93 | | | 0.96 | |  | | 2.79 | | 1.02 | |  |
| Mathematics | 2.99 | 0.99 |  | | 2.88 | | | 1.01 | |  | | 2.78 | | 0.97 | |  |
| Self-concept |  |  |  | |  | | |  | |  | |  | |  | |  |
| German | 3.30 | 0.94 |  | | 3.25 | | | 0.90 | |  | | 3.19 | | 0.97 | |  |
| English | 3.48 | 1.01 |  | | 3.41 | | | 1.00 | |  | | 3.35 | | 1.04 | |  |
| French | 3.29 | 1.08 |  | | 3.16 | | | 1.05 | |  | | 3.03 | | 1.09 | |  |
| Mathematics | 3.06 | 1.12 |  | | 2.91 | | | 1.14 | |  | | 2.85 | | 1.09 | |  |
| Career Orientation: Approach | | | |  | | |  | |  | |  | |  | |  |  |
| German |  |  |  | |  | | |  | |  | | 3.20 | | 0.97 | |  |
| English |  |  |  | |  | | |  | |  | | 3.53 | | 0.91 | |  |
| French |  |  |  | |  | | |  | |  | | 2.28 | | 0.87 | |  |
| Mathematics |  |  |  | |  | | |  | |  | | 2.45 | | 1.09 | |  |
| Career Orientation: Approach | | | | | |  |  |  |  |  |  |  |  |  |  |  |
| German |  |  |  | |  | | |  | |  | | 2.47 | | 0.85 | |  |
| English |  |  |  | |  | | |  | |  | | 1.90 | | 0.90 | |  |
| French |  |  |  | |  | | |  | |  | | 2.82 | | 1.07 | |  |
| Mathematics |  |  |  | |  | | |  | |  | | 2.86 | | 1.15 | |  |

*Note*. *N* = 161 students based on 100 multiply imputed data.

Table S3

*Convergence Between Aggregated State and Trait: Correlations Between Aggregated State and Their Corresponding Trait Emotion For Each Domain and Wave*

|  |  | Correlations | | |
| --- | --- | --- | --- | --- |
|  |  | T1 | T2 | T3 |
| Enjoyment | German | **0.311** | **0.332** | **0.229** |
|  | English | 0.152 | **0.346** | **0.261** |
|  | French | **0.454** | **0.412** | **0.266** |
|  | Mathematics | **0.336** | **0.365** | 0.161 |
| Anger | German | **0.228** | **0.307** | **0.312** |
|  | English | **0.237** | **0.265** | **0.353** |
|  | French | **0.278** | **0.373** | **0.425** |
|  | Mathematics | **0.336** | **0.343** | **0.259** |
| Pride | German | **0.408** | **0.280** | **0.472** |
|  | English | **0.323** | **0.362** | **0.414** |
|  | French | **0.420** | **0.263** | **0.298** |
|  | Mathematics | **0.352** | **0.307** | **0.249** |
| Anxiety | German | **0.359** | **0.243** | 0.202 |
|  | English | **0.212** | **0.365** | 0.209 |
|  | French | **0.349** | **0.445** | **0.520** |
|  | Mathematics | **0.280** | **0.449** | **0.467** |
| Shame | German | 0.053 | 0.191 | 0.072 |
|  | English | 0.083 | **0.310** | **0.410** |
|  | French | 0.040 | 0.181 | 0.147 |
|  | Mathematics | **0.363** | **0.234** | **0.304** |
| Boredom | German | **0.465** | **0.583** | **0.524** |
|  | English | **0.459** | **0.329** | **0.366** |
|  | French | **0.538** | **0.365** | **0.509** |
|  | Mathematics | **0.460** | **0.482** | **0.437** |

*Note*. *N* = 161 students based on 100 multiply imputed data; statistically significant correlations at *p* < .05 are in boldface.

Table S4

*Stability over Time: Auto-Correlations of Aggregated States and Traits Over One- and Two-year Spans According to Domains and Waves*

| Emotion | Domain |  | T1  🡪  T2 | T1  🡪  T3 | T2  🡪  T3 |
| --- | --- | --- | --- | --- | --- |
| Joy | German | Agg. State | **0.271** | 0.159 | **0.366** |
|  |  | Trait | **0.412** | **0.412** | **0.308** |
|  | English | Agg. State | **0.202** | 0.093 | **0.270** |
|  |  | Trait | **0.348** | **0.255** | **0.412** |
|  | French | Agg. State | **0.343** | **0.409** | **0.428** |
|  |  | Trait | **0.361** | **0.210** | **0.330** |
|  | Mathematics | Agg. State | **0.232** | **0.211** | **0.308** |
|  |  | Trait | **0.445** | 0.179 | **0.402** |
| Anger | German | Agg. State | **0.322** | **0.348** | **0.273** |
|  |  | Trait | **0.316** | **0.316** | **0.464** |
|  | English | Agg. State | **0.190** | 0.091 | **0.333** |
|  |  | Trait | **0.292** | **0.245** | **0.428** |
|  | French | Agg. State | **0.242** | 0.130 | **0.281** |
|  |  | Trait | **0.416** | **0.265** | **0.331** |
|  | Mathematics | Agg. State | **0.238** | **0.247** | **0.312** |
|  |  | Trait | **0.394** | **0.215** | **0.407** |
| Pride | German | Agg. State | **0.416** | **0.373** | **0.390** |
|  |  | Trait | **0.350** | **0.350** | **0.418** |
|  | English | Agg. State | **0.399** | **0.334** | **0.363** |
|  |  | Trait | **0.371** | **0.420** | **0.539** |
|  | French | Agg. State | **0.290** | **0.319** | **0.245** |
|  |  | Trait | **0.473** | **0.277** | **0.417** |
|  | Mathematics | Agg. State | **0.249** | **0.298** | **0.443** |
|  |  | Trait | **0.391** | **0.322** | **0.506** |
| Anxiety | German | Agg. State | **0.249** | 0.168 | **0.367** |
|  |  | Trait | **0.354** | **0.354** | **0.252** |
|  | English | Agg. State | **0.222** | **0.222** | **0.284** |
|  |  | Trait | **0.298** | **0.249** | **0.642** |
|  | French | Agg. State | **0.354** | **0.253** | **0.257** |
|  |  | Trait | **0.556** | **0.415** | **0.311** |
|  | Mathematics | Agg. State | **0.320** | **0.234** | **0.387** |
|  |  | Trait | **0.303** | **0.267** | **0.665** |

| Table S4 continued | | | | | |
| --- | --- | --- | --- | --- | --- |
| Emotion | Domain |  | Wave 1 🡪  Wave 2 | Wave 1 🡪  Wave 3 | Wave 2  🡪  Wave 3 |
| Shame | German | Agg. State | **0.223** | **0.266** | **0.407** |
|  |  | Trait | **0.445** | **0.445** | **0.377** |
|  | English | Agg. State | 0.151 | 0.174 | **0.272** |
|  |  | Trait | **0.225** | **0.199** | **0.457** |
|  | French | Agg. State | 0.089 | 0.163 | 0.170 |
|  |  | Trait | **0.319** | **0.317** | **0.362** |
|  | Mathematics | Agg. State | **0.322** | **0.241** | 0.196 |
|  |  | Trait | **0.244** | **0.254** | **0.547** |
| Boredom | German | Agg. State | **0.403** | **0.293** | **0.484** |
|  |  | Trait | **0.438** | **0.438** | **0.531** |
|  | English | Agg. State | **0.454** | **0.309** | **0.435** |
|  |  | Trait | **0.404** | **0.337** | **0.431** |
|  | French | Agg. State | **0.426** | **0.258** | **0.235** |
|  |  | Trait | **0.412** | **0.451** | **0.478** |
|  | Mathematics | Agg. State | **0.398** | **0.309** | **0.353** |
|  |  | Trait | **0.367** | **0.308** | **0.421** |

*Note. N* = 161 students based on 100 multiply imputed data; statistically significant correlations at *p* < .05 are in boldface.

Table S5

*Semi-partial Correlations of Aggregated States and Traits with Grade, Intrinsic Value, Self-concept and (Approach, Avoidance) Career Orientations*

| Emotion | Domain |  | Wave 1 | | |  | Wave 2 | | |  | Wave3 | | | | |
| --- | --- | --- | --- | --- | --- | --- | --- | --- | --- | --- | --- | --- | --- | --- | --- |
|  |  |  | Grade | Intrinsic value | Self-concept |  | Grade | Intrinsic value | Self-concept |  | Grade | Intrinsic value | Self-concept | Career Orientation | |
|  |  |  |  |  |  |  |  |  |  |  |  |  |  | Approach | Avoidance |
| Enjoyment | German | Traits | 0.23 | **0.43** | 0.25 |  | 0.11 | **0.62** | 0.26 |  | 0.20 | **0.50** | 0.31 | 0.33 | -0.18 |
|  |  | Agg. states | 0.06 | 0.02 | 0.05 |  | 0.13 | 0.05 | 0.07 |  | -0.01 | 0.05 | -0.01 | 0.03 | -0.13 |
|  | English | Traits | 0.23 | **0.48** | 0.36 |  | 0.32 | **0.49** | 0.34 |  | 0.28 | 0.46 | 0.37 | 0.19 | -0.25 |
|  |  | Agg. states | -0.08 | 0.00 | -0.05 |  | 0.00 | 0.04 | -0.01 |  | 0.01 | 0.05 | 0.01 | 0.12 | -0.09 |
|  | French | Traits | 0.35 | **0.65** | **0.48** |  | **0.23** | **0.46** | **0.39** |  | 0.27 | **0.47** | **0.37** | 0.30 | -0.27 |
|  |  | Agg. states | 0.06 | -0.02 | 0.06 |  | 0.10 | 0.08 | **0.10** |  | 0.11 | 0.22 | 0.16 | **0.24** | -0.29 |
|  | Mathematics | Traits | 0.33 | **0.62** | 0.50 |  | 0.35 | 0.46 | 0.33 |  | 0.38 | 0.46 | 0.54 | 0.38 | **-0.34** |
|  |  | Agg. states | 0.01 | 0.07 | 0.09 |  | -0.05 | 0.10 | 0.03 |  | 0.02 | 0.11 | 0.03 | 0.15 | -0.09 |
| Anger | German | Traits | -0.11 | -0.15 | **-0.18** |  | -0.11 | **-0.23** | -0.11 |  | -0.01 | -0.17 | -0.04 | -0.02 | 0.02 |
|  |  | Agg. states | -0.10 | -0.10 | -0.02 |  | -0.12 | **-0.20** | -0.07 |  | -0.04 | -0.04 | -0.06 | -0.07 | 0.09 |
|  | English | Traits | **-0.19** | **-0.36** | **-0.17** |  | -0.19 | -0.15 | **-0.21** |  | -0.13 | **-0.25** | -0.19 | -0.19 | **0.34** |
|  |  | Agg. states | 0.12 | 0.09 | **0.18** |  | -0.01 | -0.06 | 0.05 |  | 0.03 | 0.00 | 0.01 | 0.13 | -0.09 |
|  | French | Traits | **-0.31** | **-0.36** | **-0.37** |  | **-0.31** | -0.19 | **-0.21** |  | -0.19 | **-0.31** | **-0.33** | -0.21 | **0.25** |
|  |  | Agg. states | **0.16** | 0.08 | **0.20** |  | -0.07 | -0.16 | -0.15 |  | -0.07 | -0.05 | -0.02 | -0.16 | 0.11 |
|  | Mathematics | Traits | **-0.24** | **-0.24** | **-0.37** |  | **-0.20** | **-0.19** | **-0.26** |  | -0.19 | **-0.22** | **-0.23** | -0.19 | **0.26** |
|  |  | Agg. states | 0.00 | -0.08 | -0.05 |  | -0.07 | -0.08 | -0.08 |  | 0.00 | -0.08 | -0.01 | -0.09 | 0.10 |
| Pride | German | Traits | -0.02 | 0.14 | 0.09 |  | **0.23** | **0.43** | **0.35** |  | **0.24** | **0.33** | **0.22** | 0.22 | -0.11 |
|  |  | Agg. states | 0.07 | 0.02 | 0.15 |  | 0.00 | 0.00 | 0.05 |  | 0.01 | 0.07 | 0.01 | -0.02 | -0.05 |
|  | English | Traits | **0.23** | **0.31** | **0.36** |  | 0.17 | **0.29** | **0.32** |  | **0.27** | **0.31** | **0.35** | 0.20 | **-0.29** |
|  |  | Agg. states | 0.08 | -0.01 | 0.12 |  | 0.09 | 0.08 | 0.15 |  | 0.05 | -0.04 | 0.02 | 0.00 | 0.06 |
|  | French | Traits | **0.24** | **0.38** | **0.37** |  | **0.23** | **0.32** | **0.35** |  | **0.25** | **0.32** | **0.36** | 0.17 | -0.19 |
|  |  | Agg. states | 0.02 | -0.01 | 0.02 |  | -0.01 | 0.05 | -0.02 |  | -0.01 | 0.13 | 0.07 | 0.14 | -0.14 |
|  | Mathematics | Traits | **0.35** | **0.43** | **0.45** |  | **0.32** | **0.36** | **0.32** |  | **0.31** | **0.27** | **0.39** | **0.29** | **-0.31** |
|  |  | Agg. states | -0.10 | 0.08 | -0.01 |  | 0.00 | 0.16 | 0.10 |  | -0.11 | 0.08 | -0.04 | 0.04 | -0.02 |

| Table S5 continued | | | | | | | | | | | | | | | |
| --- | --- | --- | --- | --- | --- | --- | --- | --- | --- | --- | --- | --- | --- | --- | --- |
| Emotion | Domain |  | Wave 1 | | |  | Wave 2 | | |  | Wave3 | | | | |
|  |  |  | Grade | Intrinsic value | Self-concept |  | Grade | Intrinsic value | Self-concept |  | Grade | Intrinsic value | Self-concept | Career Orientation | |
|  |  |  |  |  |  |  |  |  |  |  |  |  |  | Approach | Avoidance |
| Anxiety | German | Traits | -0.04 | -0.03 | -0.04 |  | -0.01 | 0.14 | -0.03 |  | -0.04 | 0.09 | -0.01 | -0.05 | 0.08 |
|  |  | Agg. states | 0.06 | 0.03 | 0.04 |  | -0.04 | -0.08 | -0.09 |  | 0.12 | 0.00 | 0.05 | 0.02 | -0.06 |
|  | English | Traits | -0.14 | **-0.33** | **-0.27** |  | -0.19 | **-0.22** | **-0.26** |  | -0.09 | -0.08 | -0.18 | -0.16 | **0.25** |
|  |  | Agg. states | 0.04 | **0.16** | **0.20** |  | -0.01 | 0.05 | 0.12 |  | 0.06 | -0.06 | 0.03 | -0.08 | 0.14 |
|  | French | Traits | **-0.20** | -0.11 | **-0.22** |  | -0.08 | -0.08 | -0.01 |  | -0.19 | -0.06 | **-0.30** | -0.13 | **0.25** |
|  |  | Agg. states | 0.09 | 0.14 | 0.14 |  | -0.07 | -0.06 | -0.12 |  | 0.08 | -0.07 | 0.08 | -0.03 | 0.00 |
|  | Mathematics | Traits | **-0.23** | -0.16 | **-0.36** |  | **-0.28** | -0.18 | **-0.30** |  | -0.17 | -0.13 | -0.10 | -0.15 | 0.18 |
|  |  | Agg. states | -0.05 | -0.04 | -0.09 |  | 0.05 | -0.01 | -0.02 |  | -0.05 | -0.08 | -0.12 | -0.12 | 0.19 |
| Shame | German | Traits | -0.12 | -0.03 | -0.09 |  | -0.07 | 0.09 | -0.09 |  | -0.04 | 0.05 | -0.02 | 0.00 | 0.06 |
|  |  | Agg. states | 0.13 | -0.01 | 0.07 |  | 0.01 | -0.02 | -0.01 |  | 0.01 | -0.07 | -0.06 | -0.10 | **0.11** |
|  | English | Traits | -0.06 | -0.12 | **-0.18** |  | **-0.23** | **-0.27** | **-0.28** |  | -0.12 | -0.08 | -0.11 | **-0.32** | **0.41** |
|  |  | Agg. states | 0.03 | -0.01 | 0.10 |  | 0.05 | 0.06 | 0.12 |  | 0.09 | 0.00 | 0.03 | 0.03 | 0.07 |
|  | French | Traits | -0.13 | -0.01 | -0.14 |  | -0.15 | -0.02 | -0.05 |  | -0.19 | -0.08 | **-0.22** | -0.13 | **0.32** |
|  |  | Agg. states | 0.05 | 0.09 | 0.15 |  | -0.03 | -0.07 | -0.17 |  | 0.00 | -0.01 | 0.00 | -0.06 | 0.05 |
|  | Mathematics | Traits | -0.11 | **-0.20** | **-0.18** |  | **-0.29** | -0.15 | **-0.34** |  | -0.21 | -0.08 | -0.01 | -0.10 | 0.16 |
|  |  | Agg. states | -0.04 | **0.18** | -0.09 |  | 0.01 | -0.04 | -0.03 |  | -0.10 | -0.10 | -0.21 | -0.10 | 0.18 |
| Boredom | German | Traits | -0.10 | **-0.24** | -0.13 |  | -0.07 | **-0.35** | 0.01 |  | 0.04 | **-0.31** | 0.00 | -0.04 | -0.06 |
|  |  | Agg. states | -0.03 | -0.15 | 0.05 |  | -0.05 | -0.18 | -0.08 |  | -0.03 | 0.03 | -0.02 | -0.08 | 0.18 |
|  | English | Traits | 0.03 | **-0.21** | -0.03 |  | -0.06 | **-0.23** | -0.04 |  | -0.08 | -0.19 | -0.16 | -0.09 | 0.11 |
|  |  | Agg. states | 0.08 | 0.07 | 0.18 |  | 0.06 | 0.10 | 0.07 |  | 0.06 | 0.02 | 0.08 | 0.01 | 0.08 |
|  | French | Traits | -0.18 | **-0.39** | **-0.20** |  | **-0.29** | **-0.23** | **-0.21** |  | -0.15 | **-0.29** | **-0.24** | -0.20 | 0.20 |
|  |  | Agg. states | -0.01 | -0.07 | 0.04 |  | -0.12 | -0.17 | **-0.25** |  | -0.10 | -0.13 | -0.03 | **-0.24** | 0.15 |
|  | Mathematics | Traits | -0.17 | **-0.28** | -0.15 |  | -0.02 | -0.14 | -0.07 |  | -0.16 | -0.17 | -0.16 | -0.15 | **0.24** |
|  |  | Agg. states | 0.01 | -0.14 | -0.01 |  | -0.12 | -0.14 | -0.07 |  | -0.04 | -0.19 | -0.10 | -0.09 | 0.12 |

*Note*. *N* = 161 students based on 100 multiply imputed data; Semi-partial correlations of traits with external criteria were corrected for aggregated states and vice versa. Bold values signify statistically significant semi-partial correlations at *p* < .05.

Table S6

*State Aggregated Across All Three Waves with Wave-Specific and Aggregated Trait Scores*

|  | Correlations of aggregated states across waves with their respective traits | | | |
| --- | --- | --- | --- | --- |
|  | Wave-Specific Traits Scores | | | Aggregated Trait Scores |
|  | T1 | T2 | T3 |  |
| Enjoyment | .393 | .407 | .309 | .441 |
| Anger | .375 | .406 | .384 | .477 |
| Pride | .505 | .473 | .514 | .598 |
| Anxiety | .432 | .358 | .381 | .519 |
| Shame | .257 | .316 | .388 | .388 |
| Boredom | .598 | .577 | .683 | .683 |

Note. *N* = 161 students based on 100 multiply imputed data. All correlations are statistically significant at *p* < .05.
